# Isolation and characterization of *Novosphingobium oxfordense* sp. nov. and *Novosphingobium mississippiense* sp. nov. from soil, with LC-MS/MS and genome-based investigation of their glycosphingolipid production

**DOI:** 10.3389/fmicb.2026.1862985

**Published:** 2026-06-16

**Authors:** Tahir Ali, Paul D. Boudreau

**Affiliations:** Department of BioMolecular Sciences, School of Pharmacy, University of Mississippi, Oxford, MS, United States

**Keywords:** glycosphingolipids, glycosyltransferase, LC-MS/MS lipidomics, *Novosphingobium*, sequence similarity networking

## Abstract

Members of the genus *Novosphingobium* are well known for the biosynthesis of glycosphingolipids. Here, we are reporting two recently discovered species belonging to this genus, which were isolated from a potted tomato plant in Oxford, Mississippi, USA. The cells of both strains grew aerobically, were rod-shaped, and formed yellow colonies. According to the 16S rRNA gene and whole genome-based phylogeny, both strains have the highest similarity to *N. clariflavum* 164^*T*^. Genomes of both strains had a G + C content of 64 mol %, and assemblies with four circular contigs: two plasmids and two larger sequences over a megabase long. The analysis of the ortho Average Nucleotide Identity scores, along with digital DNA-DNA hybridization values, demonstrated that the two strains are distinct and unique species. Comparative genomic analysis with the reference strains of the genus *Novosphingobium* showed that the central metabolic pathways of these strains appeared to be similar, with slight variation consistent with prior reports of strains within the genus. Using MS/MS fragmentation data, glycosphingolipids with a hexuronic acid head-group could be annotated. A Sequence Similarity Network of the glycosyltransferase genes within these organisms showed a homolog of the known bacterial gene responsible for adding glucuronic acid to ceramide, consistent with the MS/MS analysis. These strains' major isoprenoid quinone was Q-10, and their predominant polyamine was spermidine. The phenotypic, chemotaxonomic, and genotypic features of strains BL-8A and BL-8H lead to the conclusion that these are two novel species of the genus *Novosphingobium*, for which the names *Novosphingobium oxfordense* (type strain = BL-8A^*T*^ = NRRL B-65725^*T*^ = LMG 34032^*T*^ = ATCC TSD-464^*T*^) and *Novosphingobium mississippiense* (type strain = BL-8H^*T*^ = NRRL B-65726^*T*^ = LMG 34033^*T*^ = ATCC TSD-465^*T*^) are proposed.

## 1 Introduction

Originally, species within the *Novosphingobium* genus were classified under the genus *Sphingomonas*; in 2001, this classification was revised, resulting in the creation of three new genera: *Sphingobium, Novosphingobium*, and *Sphingopyxis* (Takeuchi et al., 2001; Hördt et al., 2020). This proposal was based on phenotypic, chemotaxonomic, and phylogenetic analyses, which supported transferring several species from *Sphingomonas* to these new genera (Takeuchi et al., 2001; Hördt et al., 2020). These genera now constitute the *Sphingomonadaceae*, a family that belongs to the class of *Alphaproteobacteria*. Members of this family predominantly contain 2'-hydroxymyristoyl dihydrosphingosine-1-glucuronic acid (SGL-1) as the primary glycosphingolipid (GSL), a single sugar elaborated sphingolipid (SL), in their cells (Glaeser and Kämpfer, 2014). Currently, the List of Prokaryotic Names with Standing in Nomenclature has 85 child taxa in the genus *Novosphingobium*, 68 of with validly published and correct names, the type species is *N. capsulatum*, and the genus continues to grow with new work, including this report (Parte et al., 2020; List of Prokaryotic Names with Standing in Nomenclature, 2025). *Novosphingobium* species have been found in various environments, including soil, marine habitats, hot springs, wastewater treatment plants/activated sludge, freshwater, and plant rhizospheres (Niharika et al., 2013; Kämpfer et al., 2015; Chen et al., 2020; Belmok et al., 2023). Additionally, *Novosphingobium* strains have been isolated from clinical specimens associated with colorectal and biliary tract cancer (Selmi et al., 2003; Karwowska et al., 2020; Zhou et al., 2023).

In terms of morphology, bacteria belonging to the genus *Novosphingobium* are identified as yellow or whitish brown colonies, Gram-negative, non-spore-forming, and rod-shaped organisms (Takeuchi et al., 2001). They can be either non-motile or motile (depending on the presence or absence of a flagella), and grow in aerobic conditions, obtaining their energy from organic compounds, making them chemoorganotrophs (Chaudhary and Kim, 2016; Sha et al., 2017; Hördt et al., 2020).

Chemotaxonomically, *Novosphingobium* species usually have a high percentage of unsaturated fatty acids (Takeuchi et al., 2001; Nguyen et al., 2016). Certain strains of *Novosphingobium* have the ability to convert nitrate into nitrite (Takeuchi et al., 2001; Kämpfer et al., 2015). The major ubiquinone present is Q-10, while the major polyamine found in *Novosphingobium* bacteria is spermidine (Liu et al., 2021; Yoo et al., 2021; Huang et al., 2023).

Genetically, *Novosphingobium* species typically have a circular chromosome ranging from 3.12 to 6.95 Mb in size, with a G + C content of 61.5–68.8 mol % (Liu et al., 2021). The genomes of *Novosphingobium* strains typically contain roughly 3,000 to 6,000 protein-coding genes and up to 61 tRNA genes (Liu et al., 2021). Whole genome sequencing shows that *Novosphingobium* genomes often have several plasmids (Liu et al., 2021), however, there are also several genomes in this genus on GenBank which show the unusual organization of the bacterial genome across two large circular contigs, both bearing copies of core housekeeping genes (e.g., the ribosomal rRNA genes), as was reported with *Novosphingobium* sp. P6W or *Novosphingobium* sp. BL-52-GroH (Gogoleva et al., 2019; Seid and Boudreau, 2025). Comparative genomic analysis has revealed that the *Novosphingobium* genus has a high degree of genome plasticity, which allows for the adaptation to diverse environments (Wang et al., 2018). For instance, *Novosphingobium* can utilize a range of carbon sources, including carbohydrates, amino acids, and aromatic compounds (Liu et al., 2021). This adaptability is facilitated by the presence of numerous transporters and enzymes involved in the catabolism of these compounds (Strabala et al., 2012; Chen et al., 2021). Several genomes of *Novosphingobium* genus bear genes involved in the degradation of organic compounds, including xenobiotic compounds, such as polycyclic aromatic hydrocarbons and herbicides (Strabala et al., 2012; Segura et al., 2017).

One reason for interest in this genus is that *Novosphingobium* spp. synthesize GSLs, which are important components of their cell membrane (Takeuchi et al., 2001; Liu et al., 2021). GSLs are amphiphilic molecules comprised of a hydrophobic ceramide and a hydrophilic sugar component, which are typically found in eukaryotes, but have also been observed to be present in bacteria (Degroote et al., 2004; Okino et al., 2020; Guo, 2022). It has been shown that bacterial GSLs, including α-galactouronoylceramide and α-glucuronosylceramide can serve as ligands for the CD1d receptor of an antigen presenting cell, thus having an immunomodulatory effect (Morita et al., 1995; Kinjo et al., 2005). Extensive research has focused on the significance of GSLs in eukaryotes, yet the physiological functions of these molecules in bacteria (including those belonging to the *Novosphingobium* genus) remain not fully understood. Despite this knowledge gap, the isolation and study of novel GSLs from the *Novosphingobium* genus to explore the immunomodulatory potential of the GSLs is an exciting research avenue. This exploration holds promise for therapeutic applications (Okuda et al., 2019).

In this research, we have characterized two novel bacterial strains, BL-8A^*T*^ and BL-8H^*T*^ belonging to the *Novosphingobium* genus. These strains were isolated from a soil sample obtained from the roots of a potted tomato plant grown in Oxford, Mississippi, USA. The complete genomes of these strains were characterized by whole genome sequencing. By utilizing a sequence similarity network, we annotated the first candidate genes for a GSL glycosyltransferase within this particular genus. Additionally, the SL profile of each strain was investigated using an LCMS/MS-based approach. The ability of BL-8A^*T*^ and BL-8H^*T*^ to produce glycosphingolipids highlights their relevance in biomedical research. Upon morphological, chemotaxonomic and genomic characterization, these strains were identified as two new species, *Novosphingobium oxfordense* (type strain = BL-8A^*T*^) and *Novosphingobium mississippiense* (type strain = BL-8H^*T*^).

## 2 Materials and methods

### 2.1 Isolation of the bacterial strains and culture conditions

We collected a soil sample from a potted tomato plant grown in Oxford (Mississippi, USA) using a sterile Falcon tube. The sample was subsequently suspended in minimal sterile water and diluted 100 × in sterile water. Next, 50 μL of the diluted samples were spread onto agar plates of our lab's Defined Medium for Siderophores (DMS) agar plates (Crumpler, 2023). Briefly, these plates contain 0.30 g/L KH_2_PO_4_, 0.30 g/L MgSO_4_, 1.60 mL/L pyruvic acid, 2.00 g/L L-glutamine, and 2.00 g/L 3-(*N*-morpholino) propanesulfonic acid (MOPS) in ca. 80% of the total volume of deionized water, then after adjusting the pH to 7.5 with 1.0 M NaOH (aq), the mixture was diluted to the full volume with sterile water and agar was added (15 g/L if needed). After autoclaving with a 15-min liquid cycle at 121 °C, the mixture was cooled and nystatin was added to a final concentration of 50 mg/L was added before pouring. After plating the bacteria, morphologically distinct colonies were picked over several days of growth at 28 °C and purified by sub-culturing them on fresh DMS plates until pure cultures of BL-8A^*T*^ and BL-8H^*T*^ were obtained. Strains were preliminarily identified by 16S gene sequencing with a commercial vendor (GENEWIZ; South Plainfield, NJ, USA). While near-complete (≥1,400 bp) 16S amplicon sequences, prepared with the previously published 1492r and *Sphingomonadales*-specific 27f primers (Frank et al., 2008), were sequenced with a commercial vendor (Plasmidsaurus; Louisville, KY, USA) to confirm stock identities (see Supplementary material for PCR method). Afterwards, these isolates were cultured on Luria Bertani (LB) (premixed LB Broth Lennox from Sigma Life Science) plates at 28 °C. In this medium, both strains exhibited significant growth within a span of 2 days. Pure liquid cultures were supplemented 1:1 with 50% (v/v) glycerol and stored at -70 °C. Strains BL-8A^*T*^ and BL-8H^*T*^ have been deposited into the American Type Culture Collection (ATCC; Manassas, VA, USA) with the type strain deposit numbers TSD-464^*T*^ for BL-8A^*T*^ and TSD-465^*T*^ for BL-8H^*T*^; as well as with the Agricultural Research Service (NRRL; Peoria, IL, USA) under the collection numbers B-65725^*T*^ for BL-8A^*T*^ and B-65726^*T*^ for BL-8H^*T*^. The strains were also deposited with the Belgian Coordinated Collection of Microorganisms (BCCM/LMG; Ghent, Belgium) under the collection numbers LMG 34032^*T*^ for BL-8A^*T*^ and LMG 34033^*T*^ for BL-8H^*T*^.

### 2.2 Isolation of high molecular weight (HMW) DNA and *de novo* genome assembly

For our in-house genome assembly experiments, we started with isolation of the HMW DNA from BL-8A^*T*^ and BL-8H^*T*^ strains using a NucleoBond HMW DNA kit (Macherey-Nagel; Allentown, PA, USA) as per the manufacturer's protocol. The NucleoBond HMW DNA kit is specifically designed to yield large amounts of ultrapure, high molecular weight DNA fragments, and has been used in similar work (Wick, 2018). To prepare the sequencing libraries for each strain, we followed the ligation sequencing kit protocol from Oxford Nanopore Technologies (ONT) and performed the sequencing on a MinION device with a flongle flowcell (FLO-FLG001). The sequencing run lasted for 72 h and used the MinKNOW software (Quick et al., 2014; Wang et al., 2021). The resulting .fast5 files were converted to .fastq format using ONT's guppy software with the appropriate super-accurate configuration file for our kit/flowcell. The Porechop tool (version 0.2.4) was used to remove adapter sequences in the basecalled reads (Wick, 2018), then the Filtlong tool (version 0.2.1) with a minimum read length of 500 bp and by quality score to keep the top 99% of reads to ensure high-quality data (Wick, 2021). Then genome assemblies were produced from this sequencing data using the Flye assembly tool (version 2.9-b1778) (Kolmogorov et al., 2019). The resulting genome sequences were further processed by an initial polishing via the Racon tool (version 1.4.10) based on mapping results of the filtered reads to the corresponding assemblies using minimap2 (version 2.17). Finally, we performed two rounds of final polishing with Medaka (version 1.7.2, Oxford Nanopore Technologies company, Oxford, UK) to produce draft assemblies (Oxford Nanopore Technology Research, 2017; Lee et al., 2021).

Moreover, we repeated whole-genome sequencing for both strains using a commercial vendor (Plasmidsaurus; Louisville, KY, USA), which also utilized the ONT platform. Upon comparing the genomic data and assemblies from both the in-house lab and the vendor, no significant differences were found. To achieve greatest depth of coverage, we used raw reads from both sources to reconstruct the final assemblies for strains BL-8A^*T*^ and BL-8H^*T*^ using the aforementioned method with the pooled reads from both sequencing runs.

### 2.3 Genome annotation and comparative analysis

The gene prediction, annotation and G + C content of the genomes BL-8A^*T*^ and BL-8H^*T*^ were performed by using the fully automated Rapid Annotation using Subsystem Technology (RAST) pipeline (Aziz et al., 2008) and analyzed by the SEED Viewer (Overbeek et al., 2014). Moreover, the genome of four reference strains: *Novosphingobium clariflavum* 164^*T*^ (DSM 103351), *Novosphingobium lindaniclasticum* (DSM 25409^*T*^), *Novosphingobium guangzhouense* DSM 32207^*T*^, and *Novosphingobium panipatense* SM16 (DSM 22890^*T*^) were downloaded from the National Center for Biotechnology Information (NCBI) (Gupta et al., 2009; Saxena et al., 2013; Sha et al., 2017; Zhang et al., 2017). These genomes were subjected to functional gene classification using RAST in order to explore their genomes in a comparative analysis with our isolated strains.

Estimates of completeness and contamination were carried out with the CheckM2 tool (version 1.1.0) run on a Linux computer with 16 threads via CheckM2's Neural Network (Specific Model) against the DIAMOND database (Chklovski et al., 2023). An initial test run matched to the values available on the CheckM2 GitHub page (Chklovski et al., 2025), while *Novosphingobium clariflavum* 164^*T*^ (GCA_026420865.1) was analyzed along with our genomes for comparison.

As part of the submission process for depositing our genomes with GenBank, the NCBI Prokaryotic Genome Annotation Pipeline was also run to provide an annotated version of the genome (using version 6.6 for the GenBank annotation and 6.8 for the RefSeq annotation). These annotations were used for reporting the rRNA and tRNA gene counts, while the glycosyltransferase analysis was carried out on the GenBank annotated proteins available with the accessions for each strain: GCA_044029435.1 for BL-8A^*T*^ and GCA_044029535.1 for BL-8H^*T*^.

### 2.4 16S rRNA gene and whole genome phylogenies

The genomes of BL-8A^*T*^ and BL-8H^*T*^ strains were uploaded in the Type Strain Genome Server (TYGS) (https://tygs.dsmz.de, accessed on March 1st, 2024) for a 16S rRNA gene and whole genome-based taxonomic analysis, as well as calculation of digital DNA-DNA hybridization (dDDH) with closely related strains (Meier-Kolthoff and Göker, 2019). The Newick file for whole genome tree was acquired from TYGS platform, and the font style was subsequently modified by using iTOL (Letunic and Bork, 2019). Additionally, we extracted the full 16S rRNA gene sequence from the genomes of both strains, all reference and annotated *Novosphingobium* genomes available on Genbank from which full length 16S rRNA genes could be extracted (as of May 13th, 2026), and the 16S rRNA gene from *Zymomonas mobilis* subsp. *mobilis* ZM4 (NC_006526). Two full-length 16S rRNA genes were also added from strains of *Novosphingobium aerophilium* that a BLAST search indicated were highly similar to our strains. These genes were aligned in Geneious (version 2025.2.2, Dotamics, Boston, MA) with MUSCLE 5.1 with the PPP algorithm. A phylogenetic tree was then constructed using the Maximum Likelihood method in MEGA 12.1.2 with the Tamura-Nei model of nucleotide substitutions (Tamura and Nei, 1993; Kumar et al., 2024). For the figure the tree with the highest log likelihood (–11,065.48) was shown, rooted to the *Zymomonas* outgroup, with percentage of replicate trees in which the associated taxa clustered together out of 8,192 bootstraps also indicated at the branch point (Felsenstein, 1985). The initial tree for the heuristic search was selected by choosing the tree with the superior log-likelihood between a Neighbor-Joining tree (Saitou and Nei, 1987) and a Maximum Parsimony tree. The Neighbor-Joining tree was generated using a matrix of pairwise distances computed with the Tamura–Nei (1993) model (Tamura and Nei, 1993). The MP tree had the shortest length among 10 MP tree searches, each performed with a randomly generated starting tree. The analytical procedure encompassed 68 nucleotide sequences with 1,507 positions in the final dataset. We also used autoMLST (v2.0), an automated web server for Multi-Locus Sequence Analysis (MLSA), to generate high-resolution species trees with its default advanced settings (automated selection of optimal organisms and outgroups (skipping steps 2 and 3), filtering inconsistent MLST genes, and a coalescent tree) (Alanjary et al., 2019; Pourmohsenin et al., 2025).

Ortho average nucleotide identity (orthoANI) scores were calculated using the OrthoANI Tool (Windows version 0.93.1) with the genome sequences of the eight closely related type strains retrieved from the NCBI database. The orthoANI tree was calculated in both directions with different values managed by averaging, with the Genome-to-Genome Distance Calculator form 2 (Lee et al., 2016). A more complete orthoANI analysis was run on a Linux computer, with eight threads using version 2.16.0+ of the BLAST database with version 1.40 of the OrthoANI cmd.jar tool (Lee et al., 2016), against all *Novosphingobium* GenBank genome .fasta files (629 total when accessed on June 30th, 2025). Subsequently, two strains of *Novosphingobium aerophilum* were published which by BLAST to the 16S rRNA gene, were highly similar to BL-8A^*T*^ and BL-8H^*T*^, another heatmap was generated with the Windows version of the tool (as above) for the most closely related strains from this analysis and the new *Novosphingobium aerophilum* strains.

### 2.5 Sequence similarity network (SSN) generation

Using the GenBank annotations, every annotation featuring a “glycosyltransferase” was extracted from the two genomes, note that none was annotated as being involved in sphingolipid biosynthesis. This was 68 protein sequences from BL-8A^*T*^ and 56 from BL-8H^*T*^. 1,559 glycosyltransferase proteins were extracted from other members of the genus *Novosphingobium*, specifically every annotated reference genome (44 total) available at the time (accessed June 16th, 2022). Previously annotated sequences were also available: Okino and coworkers published on a galactosyltransferase from *Bacteroides fragilis* NCTC 9343 (CAH08930.1) and two glucuronosyltransferases from *Sphingobium yanoikuyae* S72 (ATI80198.1) and *Z. mobilis* subsp. *mobilis* ZM4 (AAV90581.1) (Okino et al., 2020). Stankeviciute and coworkers also reported the proteins sphingolipid glycosyltransferase 1 (Sgt1) (ACL94258.1), a sphingolipid glucuronosyltransferase, that adds glucuronic acid onto ceramide, and the second protein, Sgt2 (ACL94257.2), a sphingolipid glycosyltransferase, that adds on a hexose sugar to form a disaccharide glycosphingolipid in *Caulobacter crescentus* NA1000 (Stankeviciute et al., 2019). The bifunctional glycosyltransferase from *Agrobacterium fabrum* C58 (AAK88039.2) that Okino and coworkers had also used as a reference was also included (Okino et al., 2020). A complete list of standards with protein accession numbers is provided in Supplementary Table S7. These 1,689 protein sequences were combined into a single .fasta file and submitted to the EFI-Enzyme Similarity Tool (Zallot et al., 2019), the network was created with the default settings of an *E*-value of 5 for the SSN Edge Calculation Option and then an Alignment Score setting of 35 with no filtering based on length or taxonomy, no use of neighborhood connectivity, and no exclusion of UniProt-defined fragments. To visualize the network, Cytoscape (version 3.10.4) was used (Kohl et al., 2011), note that for the final image clusters containing no hits to the references or from the type strains (those clusters with proteins only from the “Other *Novosphingobium*” group) were deleted. Individual pairwise sequence comparisons of hits from this analysis were carried out in Geneious (version 2025.2.2) with MUSCLE using the PPP algorithm.

### 2.6 Morphological, physiological, and biochemical analyses

For a comparative physiological and biochemical investigation, we used strains BL-8A^*T*^ and BL-8H^*T*^ together with two reference strains, *N. clariflavum* 164^*T*^ (DSM 103351^*T*^) and *N. guangzhouense* DSM 32207^*T*^. These known strains were procured from the Leibniz Institute DSMZ—German Collection of Microorganisms and Cell Cultures, located in Braunschweig, Germany based on the similarity observed in the initial 16S tree. All of these strains were cultured on LB agar plates and then picked into LB broth before storage as frozen culture stocks at a temperature of –70 °C in LB broth supplemented 1:1 with 50% (v/v) glycerol.

The four pure cultures were incubated in LB media at 28 °C, pH 7.0, without salt supplementation as the default. We started our analysis by changing one growth variable at a time: we grew cultures at 4, 10, 18, 27, 30, 32, and 40 °C, we added sodium chloride to LB medium at concentrations of 0, 0.05, 0.1, 0.3, 0.75, 1.5, 3.0, 3.5, 5.0, and 7.5% (w/v), and we buffered LB medium with tricine and citric acid monohydrate (each at 2 g/L) to examine growth over a pH range of 4.02, 5.05. 6.04, 7.02, 7.52, 8.03, 8.52, and 9.05. Growth was observed by measuring the optical densities (absorbance at 600 nm) after growth over these ranges to determine the optimal temperature, pH, and salt concentration for the growth of these strains as determined by maximal growth after 48 h.

Initial pH tolerance results for the reference strain *N. clariflavum* 164^*T*^ (DSM 103351^*T*^) did not agree with the original characterization of this type strain (Zhang et al., 2017), so the pH tolerance experiment was repeated with an expanded media panel. Strains were grown triplicate as above using: LB medium buffered with tricine and citric acid monohydrate, each at a higher loading of 2.5 g/L, at a pH of 4.08, 4.50, 5.04, 6.03, 6.99, 7.54, 8.01, and 9.03; as well as Tryptic Soy Broth (TSB) buffered with tricine and citric acid monohydrate (each at 2.5 g/L) at a pH of 4.00, 4.52, 5.05, 6.03, 7.04, 7.52, 8.04, and 8.98. Strains were also grown in TSB without buffer, as the original report did not use buffer (Zhang et al., 2017), over the same pH range, however, the growth without buffer was not seen to be consistent within the biological triplicates so the results without buffer were discarded.

Cell morphology was observed using field-emission scanning electron microscopy (on a JEOL JSM-7200, Tokyo, Japan). The cells for this imaging work were cultured in LB broth at 28 °C for 24 h (mid-log phase) and samples were prepared for electron microscopy using chemical drying method based on an established protocol (Koon et al., 2019). While Gram staining was performed according to the American Society of Microbiology protocol (Smith and Hussey, 2005).

To evaluate substrate utilization profiles, we modified our DMS, using 0.2% (w/v) NH_4_NO_3_ as nitrogen source instead of L-glutamine and 0.1% (w/v) of various carbon sources to replace pyruvic acid [also supplemented with 0.0005% (w/v) D-amino acids]. The organic compounds utilized as the sole carbon sources included D-glucose, glycine, pyruvic acid, D-mannose, L-arabinose, D-galactose, D-glucuronic acid, D-mannitol, D-maltose, *N*-acetyl glucosamine, citric acid monohydrate, and decanoic acid (purchased from various supplies as lab grade or better). After 2 days of cultivation, the optical density at a wavelength of 600 nm was measured to compare the growth across the carbon sources. Moreover, we also conducted biochemical analysis utilizing API 20NE strips (bioMérieux) according to the manufacturer's protocol.

We examined catalase activity through the observation of effervescence of a fresh 3% (v/v) H_2_O_2_ solution exposed to cells, whereas oxidase activity was assayed by the addition of a droplet of 1% (w/v) tetramethyl-β-phenylenediamine (TCI; Tokyo, Japan) to the culture, followed by the assessment of the resultant color change (He et al., 2022). Additionally, we performed the hydrolysis of starch, casein, tween 40, tween 80 and nitrate reduction by using previously published methods (Tindall et al., 2014).

The susceptibility of the strains to antibiotics was evaluated through the agar-diffusion technique using antibiotic-impregnated discs (Hudzicki, 2009). A bacterial suspension of 100 μL was plated onto Muller-Hilton agar medium and incubated at 28 °C for 2 days. The tested antibiotics included ampicillin (10 μg), ciprofloxacin (5 μg), chloramphenicol (30 μg), carbenicillin (100 μg), kanamycin (30 μg), streptomycin (100 μg), and tetracycline (30 μg).

### 2.7 Extraction and LC-MS/MS-based profiling of glycosphingolipids

Strains BL-8A^*T*^ and BL-8H^*T*^ were cultivated in LB broth at pH 7.0 and 28 °C for 2 days, harvested, and subjected to lipidomic extraction. GSLs and other polar lipids were extracted using chloroform-methanol (2:1) for 3 h at room temperature (Brown et al., 2019; Johnson et al., 2020). Afterwards, the extract underwent filtration, was transferred into new vials, and evaporated under vacuum. The extracted lipids were then dissolved in methanol, with water added to adjust the methanol concentration to 10%. The samples were prepared using a HyperSep C18 cartridge (100 mg, Thermo Scientific), which was conditioned with 100% methanol and then 100% water, followed by loading the sample in 10% methanol and eluting with 100% methanol (1 mL wash and elution volumes).

LC-MS/MS analysis of the putative glycosphingolipids followed prior work on this molecule class (Brown et al., 2019; Johnson et al., 2020). This analysis was conducted utilizing an Agilent 6530C Q-TOF LCMS system, which was equipped with an Agilent jet stream source and an Agilent 1260 Infinity II pump stack. Chromatographic separation was accomplished using a Phenomenex Gemini C18 HPLC column (50 mm × 2 mm dimensions, 5 μm particle size). The LC pump protocol used two solvents, solvent A: consisting of H_2_O (redistilled) with 0.5% formic acid (98.0 to 100% puriss grade, Sigma Aldrich), and solvent B: consisting of MeOH (various suppliers, always LCMS grade) with 0.5% formic acid. The gradient started with an elution of 20% solvent A and 80% solvent B for 2 min, followed by a linear gradient to 90% solvent B over 5 min, then a linear gradient to 100% solvent B over 10 min, and finally a hold at 100% solvent B for 5 min, and finally, a return to the starting elution with a re-equilibration for 3 min at a flow rate of 500 μL/min. A 10 μL injection volume was used with the autosampler. Centroid data was collected using an Auto MS/MS method, collected in positive ion mode with absolute storage thresholds of 200 and 6 for the MS and MS/MS scans, respectively. The capillary voltage was set to 4 kV, and the fragmentor voltage at 200 V. The drying gas temperature was maintained at 300 °C with a nebulizer pressure of 50 psi.

### 2.8 Molecular networking and molecular annotation

The molecular network was created using the Global Natural Products Social (GNPS) platform (Wang et al., 2016). Raw data files, obtained from the Agilent 6530C Q-TOF LCMS, were converted to an open-source format (.mzXML) prior to being uploaded to the online platform. For this network, the precursor ion mass tolerance and the MS^2^ fragment ion were set to 0.05 Da, with a minimum matched fragment ion setting of 6, a minimum cluster size setting of 2, and a cosine score setting of 0.65. Subsequently, the generated molecular networks were visualized using Cytoscape software (3.9.0) (Kohl et al., 2011). To verify the matched and unmatched SL/GSLs, MS/MS spectra were individually inspected to select diagnostic product ions essential for annotation and the differences between theoretical and experimental *m/z* values were calculated (see Supplementary Figures S1–S7 and Supplementary Table S1).

### 2.9 Respiratory quinone and polyamines analysis

Respiratory quinones were extracted and analyzed using LC-MS/MS method mentioned above for the lipidomics. However, we identified the dominant respiratory quinone using MS^1^ data alone. A separate polyamines analysis was conducted where these compounds were isolated, derivatized, and identified using by LC-MS/MS, with pure spermidine (Sigma, analytical standard) as external standard; this protocol followed a published literature method, however, we replaced 2-chlorobenzyl bromide for the original derivatizing agent (Ibarra et al., 2015).

## 3 Results and discussion

### 3.1 Morphological, physiological, and biochemical characteristics

The most favorable conditions for the growth of strains BL-8A^*T*^ and BL-8H^*T*^ were found to be at 28 °C, neutral pH, and without (or with minimal) salt supplementation (with growth impairment when supplemented above 1.5% or 0.75%, respectively). The pH tolerance for BL-8A^*T*^ and BL-8H^*T*^ were consistent in both media tested, though strain *N. clariflavum* 164^*T*^ (DSM 103351) was never observed to grow at pH 4.0 as with the original report, however that report used an unbuffered medium, which may be the result of this inconsistency (Zhang et al., 2017). Both strains were Gram-stain-negative, aerobic, non-motile, had rod-shaped cells, and formed yellowish smooth colonies on solid agar (Supplementary Figure S8). The strains could use a wide diversity of carbon sources, utilizing D-glucose, glycine, pyruvic acid, D-mannose, L-arabinose, D-galactose, D-glucuronic acid, maltose, *N*-acetyl glucosamine, citric acid, and decanoic acid. However, as with other strains in the genus evaluated, BL-8A^*T*^ and BL-8H^*T*^ did not utilize D-mannitol as a carbon source. Enzymatic differences were also evaluated among the strains; of these, strains BL-8A^*T*^ and BL-8H^*T*^ were the only ones that tested positive for catalase and nitrate reduction. The primary respiratory quinone in both BL-8A^*T*^ and BL-8H^*T*^ strains was Q10 and major polyamine in both was spermidine. Detailed differences among strains BL-8A^*T*^, BL-8H^*T*^, and the two reference strains in terms of physiological and biochemical characteristics are summarized in Table 1, while a full list of the biochemical characteristics are provided in Supplementary Table S2.

**Table 1:** Key physiological and biochemical differences of strains BL-8A^*T*^ and BL-8H^*T*^ compared to representatives of the genus.

Characteristics	1	2	3	4
DNA G + C content (mol %)	64.0	64.4	65.9	60.2
pH range for growth				
In buffered TSB	5.0–7.5	5.0–8.0	5.0–8.0	6.0–8.0
In buffered LB	5.0–7.5	5.0–8.0	5.0–7.0	5.0–8.0
Supplemental NaCl (%) range for growth	0–3.0	0–3.0	0–2.0	0–4.0
Nitrate reduction	–	+	–	–
Oxidase activity	+	+	–	+
Catalase activity	+	–	–	–
Hydrolysis of				
Tween 40	–	–	–	w
Starch	–	+	–	+
Enzyme assays				
Assimilation of *N*-acetyl-glucosamine	+	+	–	+
Assimilation of potassium gluconate	+	+	–	–
Assimilation of capric acid	w	–	–	–
Assimilation of adipic acid	w	w	–	–
Assimilation of malic acid	–	w	+	–
16.6-8.6,-13242.5ptAssimilation of trisodium citrate	–	–	–	+
Carbon source utilization				
D-mannitol	–	–	+	+
Antibiotic resistance				
Chloramphenicol	–	+	+	+

Strains: **1** = *N. oxfordense* BL-8A^*T*^, **2** = *N. mississippiense* BL-8H^*T*^, **3** = *N. clariflavum* 164^*T*^ (DSM 103351), **4** = *N. guangzhouense* DSM 32207^*T*^.

+= positive, −= negative, w = weakly positive.

Quinone Q-10 was identified through the MS^1^ data, which provided precise molecular ion [M+H]^+^ information at 863.6925 and 863.6903 (for BL-8A^*T*^ and BL-8H^*T*^, respectively; expected 863.6912 *m/z*, 1.5 ppm and –1.04 ppm, respectively) (Mamun et al., 2022), thereby confirming the presence of Q-10 in the extracts from both strains. While spermidine was identified in the extracts of both the strains using pure spermidine as an external standard for comparison.

In the antimicrobial susceptibility testing, strain BL-8A^*T*^ exhibited sensitivity to ampicillin, ciprofloxacin, chloramphenicol, carbenicillin, kanamycin, and tetracycline, while showing resistance to streptomycin. Conversely, strain BL-8H^*T*^ demonstrated sensitivity to ampicillin, ciprofloxacin, carbenicillin, kanamycin, and tetracycline, but exhibited resistance to chloramphenicol and streptomycin.

### 3.2 LCMS-based characterization of glycosphingolipids

**Figure 1:**
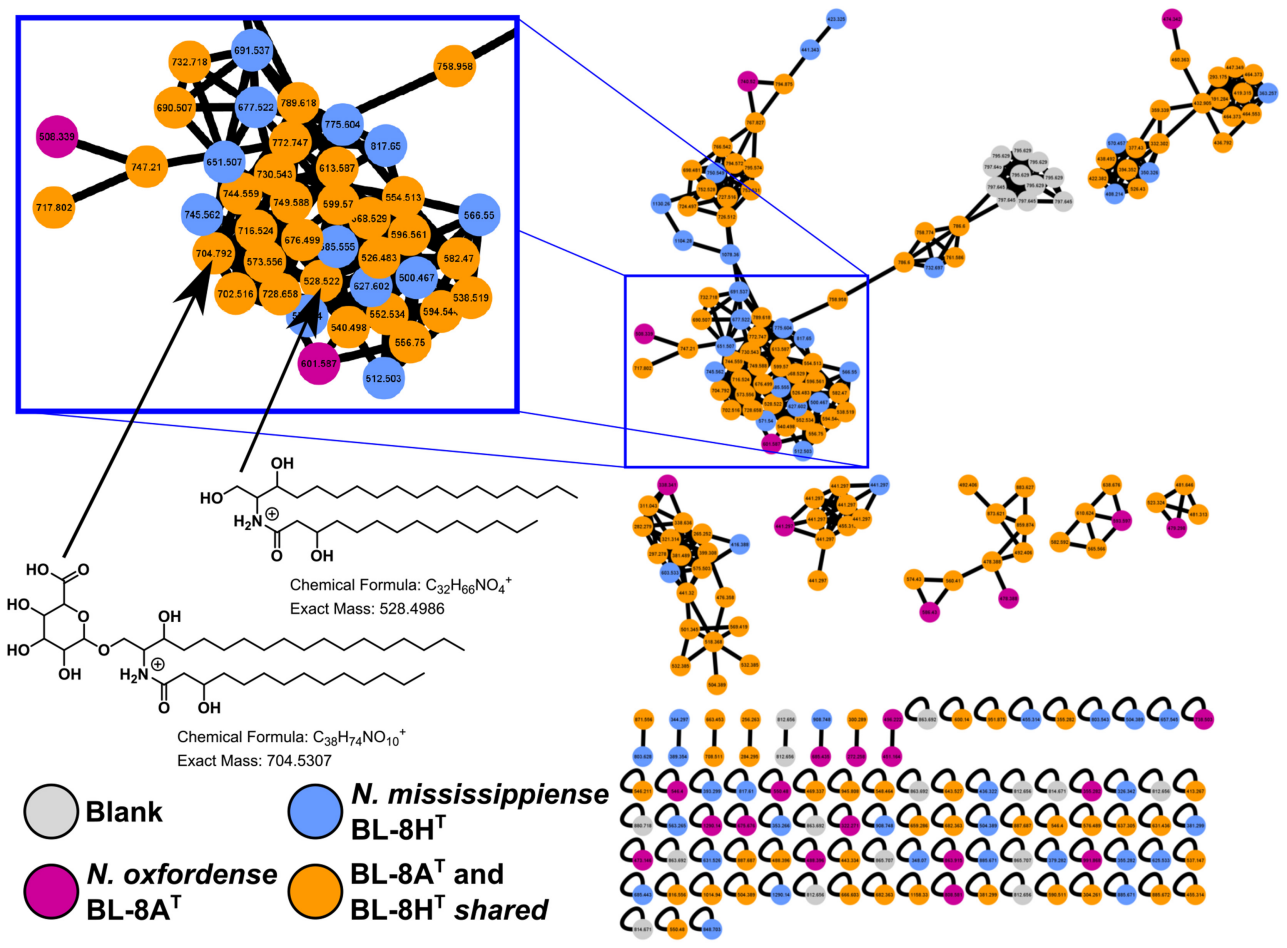
A molecular network illustrating the lipidomics data. Key masses (orange circles) shared between BL-8A^*T*^ and BL-8H^*T*^ in the sphingolipid cluster are detailed with the zoomed-in box. Two examples of annotated masses are depicted with their structures. Phosphoethanolamine lipids were also seen in this cluster (not highlighted). Adapted from Figure 3.4 published in Tahir (2025), copyright Tahir Ali 2025, source: https://egrove.olemiss.edu/etd/3500.

GSLs represent a diverse and complex lipid class, featuring a sphingoid base, usually in the form of a ceramide, as the hydrophobic lipid segment, linked to a hydrophilic glycan through a glycosidic bond (Olsen and Jantzen, 2001). A structure that fragments predictably in MS/MS fragmentation analysis, here lipid extracts from both BL-8A^*T*^ and BL-8H^*T*^ underwent LC-MS/MS analysis for a preliminary characterization of their GSLs. The acquired data were used to construct a molecular network via the GNPS tool (Wang et al., 2016). Within this network, a cluster was identified containing hits associated with phosphoethanolamine lipids and SLs. This facilitated the preliminary identification of known SLs in the bacterial extracts matching to publicly available library spectra and many unknown masses within a larger sphingolipids cluster, see Figure 1.

A manual annotation of masses within this cluster was used to match predicted ceramide and GSLs structures to the observed MS/MS spectra. With fragments representing the sphingosine base moiety, the fatty acyl side chain, and the polar sugar head group (through specific fragment ions or neutral loss) key to the analysis (see Supplementary Figures S1–S7). Notably, a neutral loss of 194 Dalton, corresponding to a hexuronic acid like glucuronic acid (GlcA), was consistent with GSLs bearing glucuronic acid in both strains. GlcA-GSLs represent the predominant GSLs found in the cell membranes of *Novosphingobium* members (Glaeser and Kämpfer, 2014). We did not conduct an analysis of the absolute configuration of the sugar from the GSLs in our strains, but hypothesize the configuration is consistent across the genus. Most of the sphingolipids exhibited similarity between BL-8A^*T*^ and BL-8H^*T*^, although a few sphingolipids differed between them. To elucidate these similarities, we conducted an annotation of sphingolipids present in both strains. Utilizing MS/MS fragmentation analysis, we annotated four peaks consistent with ceramides at 528 *m/z* [d18:0/14:0(2OH)], 554 *m/z* [d20:1/14:0(2OH)], 556 *m/z* [d18:0/16:0(2OH)], and 568 *m/z* [d21:1/14:0(2OH)] present in both of our strains (Supplementary Figures S1–S4). Additionally, three peaks consistent with one-sugar glycan glycosphingolipids, annotated at parent masses of 704 *m/z*, 730 *m/z*, and 744 *m/z*, were present in both of our strains (Supplementary Figures S5–S7). Moreover, preliminary lipidomic analysis of these strains in our lab was consistent with this result (Biggs, 2022). Future studies will involve annotating the remaining GSLs in the molecular network and confirming the absolute configuration of the sugar headgroup in the annotated GSLs as glucuronic acid.

### 3.3 Phylogenetic analysis

Analysis based on 16S rRNA gene sequence indicated that strains BL-8A^*T*^ and BL-8H^*T*^ belong to the genus *Novosphingobium*, with all the most similar reference sequences coming from that genus. A Maximum-Likelihood phylogenetic tree constructed using the genome-derived complete 16S rRNA gene sequences did not show robust clustering around BL-8A^*T*^ or BL-8H^*T*^ (Supplementary Figure S9). With BL-8A^*T*^ often clustering with two strains of *Novosphingobium aerophilium* (which notably did not clade with the type strain of that species). The 16S rRNA genes of BL-8A^*T*^ or BL-8H^*T*^ had high similarity to each other, with the closest type strain being *N. guangzhouense* SA925^*T*^ (Supplementary Figure S9). Specifically, a 99.3% pairwise identity to each other, and a 98.5% and 98.7% pairwise identity comparing *N. guangzhouense* SA925^*T*^ to strains BL-8A^*T*^ and BL-8H^*T*^, respectively. However, a separate analysis by autoMLST, using the web platform to perform an automated multi-locus sequence analysis (Alanjary et al., 2019; Pourmohsenin et al., 2025), suggested that the closest type strain to either BL-8A^*T*^ or BL-8H^*T*^ was *Novosphingobium clariflavum* 164^*T*^, with the strain *Novosphingobium* sp. KA1 also clading closely to these strains (Supplementary Figure S10), a result in keeping with the subsequent whole genome-based comparison.

### 3.4 Genome analysis

**Figure 2:**
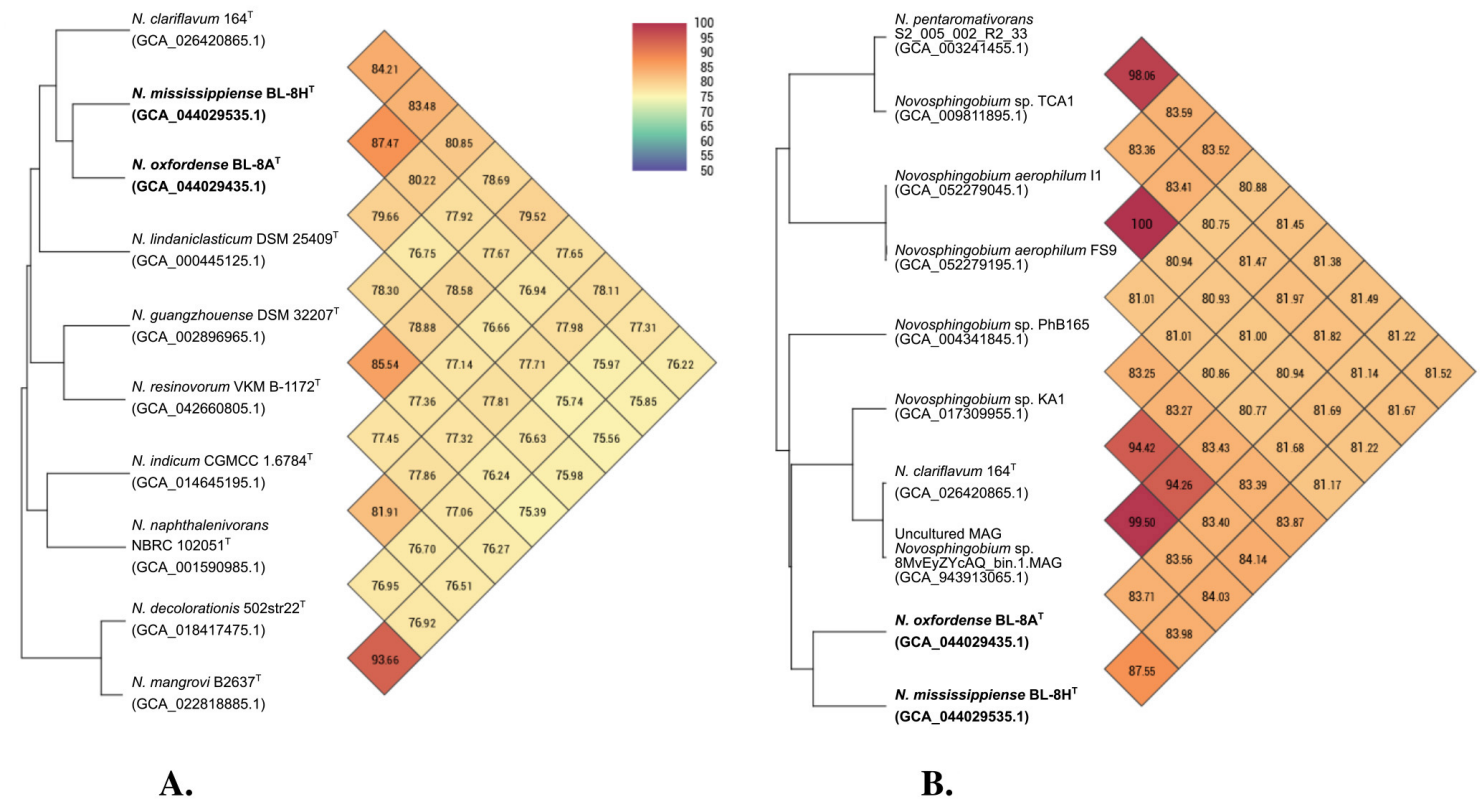
OrthoANI was calculated and heatmaps generated using the OrthoANI Tool (version 0.93.1) (Lee et al., 2016). **(A)** In comparison against eight *Novosphingobium* type strains, the new species described here (bold) consistently clade apart. **(B)** This analysis remained true when comparing to the most closely related genomes available on GenBank (accession codes in parentheses), which include undescribed strains and uncultured environmental strains. Results which support their novelty as distinct species, with < 90% score to closely related species and strains, significantly lower than the recommended threshold of 95% for ANI typically used to define distinct species (Kim et al., 2014; Meier-Kolthoff et al., 2013). **(A)** The heatmap with type strains. **(B)** The heatmap with top hits. Adapted from Figure 3.2 published in Tahir (2025), copyright Tahir Ali 2025, source: https://egrove.olemiss.edu/etd/3500.

The assembled genome of strain BL-8A^*T*^ was 7.2 Mb in length with four circular contigs, a G + C content of 64.0 mol %, an N50 of 3,930,791, and a mean coverage of 204 × , while the annotation showed 15 rRNA genes and 65 tRNA genes. The assembled genome of strain BL-8H^*T*^ was 6.1 Mb in length with four circular contigs, a G + C content of 64.4 mol %, an N50 of 3,841,346, and a mean coverage of 443 × , while the annotation showed 15 rRNA genes and 66 tRNA genes. Analysis with CheckM2 estimated the completeness of both genomes was 100%, with contamination estimated at 4.15% and 1.54% for BL-8A^*T*^ and BL-8H^*T*^, respectively; for comparison *Novosphingobium clariflavum* 164^*T*^ was estimated at 100% completeness and 1.12% contamination (Chklovski et al., 2023). These genomes have been deposited in GenBank, with accession numbers for the contigs of CP147005-CP147008 and CP147001-CP147004, for strains BL-8A^*T*^ and BL-8H^*T*^ respectively. A phylogenomic tree, based on whole genome sequences constructed using the TYGS pipeline demonstrated that these two strains clustered closely with other species of the *Novosphingobium* genus and had their nearest type strain was *N. clariflavum* 164^*T*^; it also supported the assignment of both BL-8A^*T*^ and BL-8H^*T*^ as new species (Supplementary Figure S11) (Meier-Kolthoff and Göker, 2019). OrthoANI analysis revealed that strain BL-8A^*T*^ exhibited values ranging from 75.56% to 80.85% when compared with other *Novosphingobium* type strains, whereas strain BL-8H^*T*^ showed values ranging from 75.85% to 83.48%, see Figure 2 (Lee et al., 2016). Notably, the highest OrthoANI score for BL-8A^*T*^ and BL-8H^*T*^ was with each other, even when compared against the most closely related strains on GenBank by including undescribed species, see Figure 2. Moreover, the estimated dDDH values among BL-8A^*T*^ and BL-8H^*T*^ and other *Novosphingobium* spp. fell within the range of 19.6% to 34.8% (Supplementary Table S3). These numbers are significantly lower than the recommended thresholds of 95% for ANI and 70% for dDDH, which are typically used to define distinct species (Meier-Kolthoff et al., 2013; Kim et al., 2014). Consequently, it can be inferred that both BL-8A^*T*^ and BL-8H^*T*^ represent novel species within the *Novosphingobium* genus. A more comprehensive investigation with the OrthoANI Tool showed that no *Novosphingobium* genome had ≥90% score to either of our strains (Supplementary Tables S4, S5) (Lee et al., 2016), as such we cannot include other isolates or metagenome-assembled genomes within the two species descriptions. Little valuable insight could be gained about distribution or ecological context of the strains most closely related to BL-8A^*T*^ and BL-8H^*T*^ because the most closely related strains or metagenome-assembled genomes were globally distributed (e.g., *N. clariflavum* 164^*T*^ from China, GCA_026420865.1) or were isolated from diverse environments (e.g., the uncultured *Novosphingobium* sp. metagenome-assembled genome isolate 8MvEyZYcAQ_bin.1.MAG from human skin, GCA_943913065.1).

The RAST annotation predicted 6,702 and 5,723 protein-coding sequences (CDS) for strain BL-8A^*T*^ and BL-8H^*T*^, respectively. An overview of the genomic features of these strains is presented in Supplementary Table S6 alongside four reference strains: *N. clariflavum* 164^*T*^ (DSM 103351), *N. lindaniclasticum* DSM 25409, *N. guangzhouense* DSM 32207^*T*^, and *N. panipatense* SM16 (DSM 22890). The gene compositions of these strains had meaningful patterns, all six strains had genes putatively encoding proteins associated with biotin, thiamin, heme and siroheme, coenzyme B12, riboflavin, NAD and NADP cofactor, pyridoxine (vitamin B6), folate, coenzyme F420, and coenzyme A biosynthesis. Furthermore, all strains were found to harbor genes associated with resistance to copper, fluoroquinolones, beta-lactamase, and cobalt-zinc-cadmium. With the exceptions of BL-8H^*T*^ and *N. panipatense*, the strains exhibited genes potentially encoding proteins linked to mercuric reductase. Conversely, all strains, except BL-8H^*T*^, possessed genes related to chromium tolerance. In terms of membrane transport systems, genes associated with protein secretion systems, specifically type II and IV, were present in all strains, while only BL-8A^*T*^ carried genes related to the type VII protein secretion system. None of the strains showed genes related to type I, type III, type V, type VI, or type VIII secretion systems. None of the strains exhibited genes associated with motility and chemotaxis, consistent with our analysis in soft agar. Only BL-8A^*T*^ and *N. panipatense* contained genes related to siderophore biosynthesis, while all strains possessed putative genes for the biosynthesis of auxin (see Supplementary Table S6).

### 3.5 Sequence similarity network (SSN)

**Figure 3:**
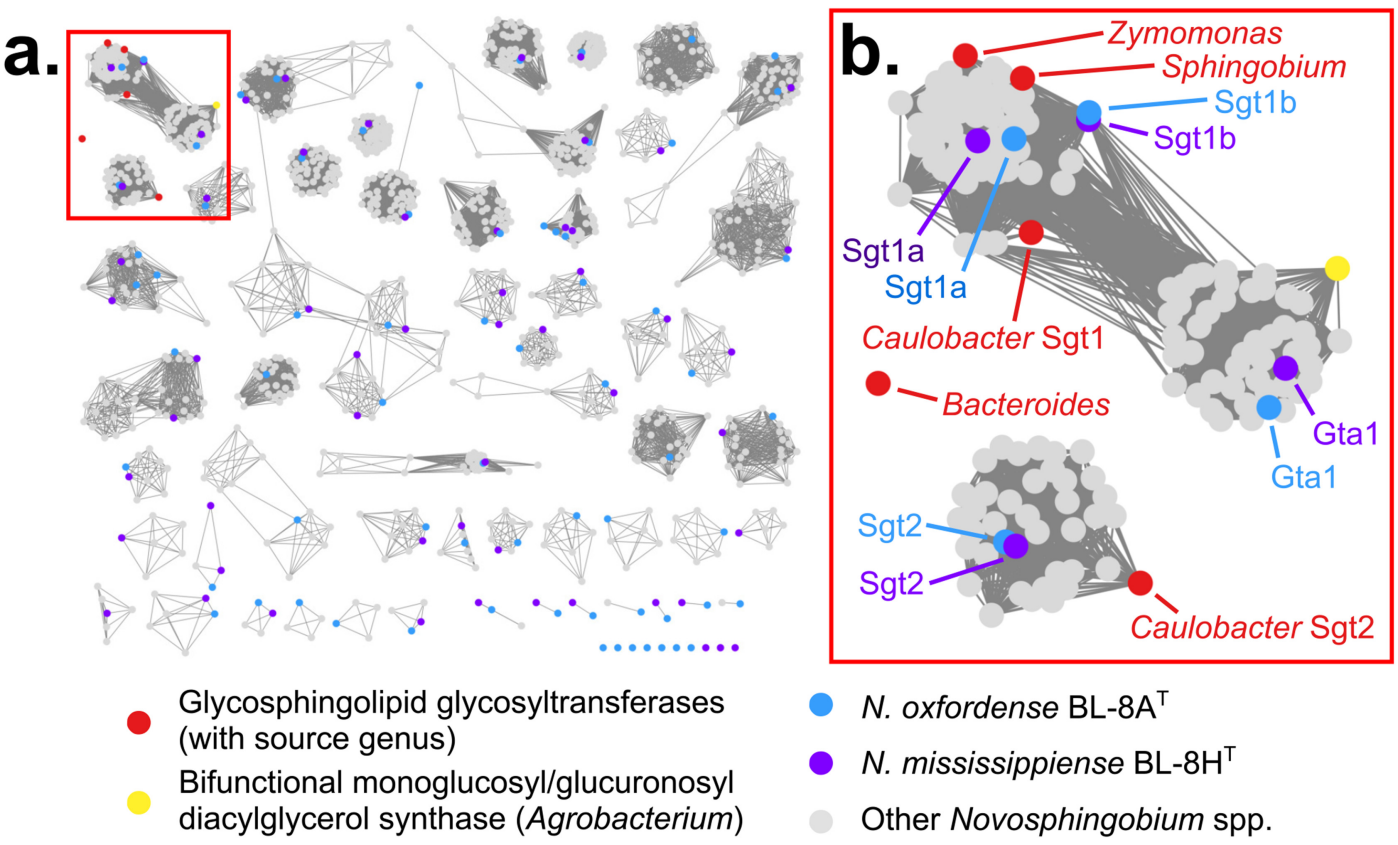
The glycosyltransferase sequence similarity network. **(a)** The network displayed clustering of proteins from the type strains with numerous other *Novosphingobium* proteins. **(b)** Three clusters, the first linked to the *Sphingomonadales* glucuronosyltransferases and Sgt1 from *Caulobacter*, the second to the *Agrobacterium* standard, and the third to Sgt2 from *Caulobacter*, red highlighted box in the full network, were of particular interest. Interestingly, the cluster with standard enzymes that add glucuronic acid to ceramide had two homologs from each type strain, indicating that these species have two copies of the Sgt1 gene, while the other clusters of homologs, for Sgt 2 and the Glycosyltransferase *Agrobacterium*-like (Gta) had only one representative each. Note that the distantly related *B. fragilis*'s glycosyltransferase standard was not observed to cluster with any of the glycosyltransferases from the *Sphingomonadales*.

The driving motivation to better understand glycosphingolipid biosynthesis necessitated a close inspection of the genes annotated as glycosyltransferases in these genomes. The putative glycosyltransferase proteins from the genome annotation were compared using the EFI-Enzyme Similarity Tool against homologs from other *Novosphingobium* and standards with known roles (see Supplementary Table S7) (Zallot et al., 2019). This network showed homologs from the type strains clustering with glucuronosyltransferases from *Zymomonas, Sphingobium*, and *Caulobacter* that are known to be involved in the biosynthesis of glucuronosylceramide (Stankeviciute et al., 2019; Okino et al., 2020), a second cluster that had the standard of the *Agrobacterium* bifunctional monoglycerol/glucuronosyl diacylglycerol synthase (Okino et al., 2020), and a third that clustered with Sgt2 from *Caulobacter* (Stankeviciute et al., 2019), see Figure 3. The homologs that clustered with the glucuronosyltransferases (e.g., Sgt1) were not a surprise, given the observation of the hexuronic-bearing glycosphingolipid observed in the lipidomic analysis. The fact that this cluster had two homologs from each type strain was surprising. A pairwise sequence comparison showed that the proteins dubbed Sgt1a were more similar to the glucuronosyltransferase homologs from the closely related *Zymomonas* and *Sphingobium* (Supplementary Table S8) (Okino et al., 2020). As such, it is hypothesized that these proteins are responsible for installing glucuronic acid onto ceramide in both of the type strains. The second copy of this gene, Sgt1b, in both strains is found on the other chromosome and has less homology to the *Sphingomonadales* glucuronosyltransferase standards (Supplementary Tables S8, S9). This may indicate that the additional copy provides redundancy, as it was more similar to Sgt1 from *Caulobacter* (Supplementary Table S8), or this homolog may install an as yet unknown novel sugar headgroup. We were unable to annotate any disaccharide glycans attached to ceramide in the lipidomic analysis, though the presence of a Sgt2 homolog in the SSN, suggests these strains may have such a biosynthetic capacity (Stankeviciute et al., 2019). Another possibility is that the putative Sgt2 proteins are involved in appending a sugar onto an non-ceramide-backed glycosphingolipid. The final annotated cluster, dubbed Gta (for Glycosyltransferase *Agrobacterium*-like), with the *Agrobacterium* bifunctional monoglycerol/glucuronosyl diacylglycerol synthase standard is unlikely to be involved in glycosphingolipid biosynthesis (Okino et al., 2020). Gene knockout work is underway in our laboratory to confirm the annotations provided via this SSN analysis and will be presented in a future publication. Protein accession numbers, genome location, sequence-based annotation, and amino acid length for each of the discussed type strain proteins can be found in the Supplementary Table S9.

## 4 Conclusion

We isolated two strains of *Novosphingobium* from the soil of a potted tomato plant. Preliminary 16S rRNA gene analysis and later genotypic comparison supported both of these strains as being novel species, with phenotypic and chemotaxonomic used to identify commonalities and differences between strains BL-8A^*T*^ and BL-8H^*T*^ and closely related type strains. The novel species have been named as *Novosphingobium oxfordense* sp. nov. and *Novosphingobium mississippiense* sp. nov., respectively.

### 4.1 Description of *Novosphingobium oxfordense* sp. nov.

*Novosphingobium oxfordense* (ox.ford.en'se. N.L. neut. adj. *oxfordense*, pertaining to Oxford, the city in Mississippi where the soil sample source of the type strain, a potted tomato plant, was grown).

Cells are Gram-stain-negative rods (1.2–1.5 μm). Colonies are yellow, circular and smooth after 48 h at 28 °C on LB. Grows at 4–40 °C (optimum, 28 °C), pH 4.0–9.5 (optimum, pH 7.5) and 0–7.5 % (w/v) NaCl supplementation to LB (optimum, 0–1.5 %). It has a 7.2 Mb genome with four circular contigs, a G + C content of 64 mol %, and 6,702 CDS available on GenBank under the accession numbers CP147005–CP147008. Oxidase and catalase-positive, while urease-negative. It utilizes D-glucose, glycine, pyruvic acid, D-mannose, L-arabinose, D-galactose, D-glucuronic acid, maltose, N-acetyl glucosamine, citric acid and decanoic acid, it did not utilize D-mannitol as carbon source. The major ceramides are 528 *m/z*, 554 *m/z*, and 568 *m/z*, with each of these ceramides also being elaborated with a hexuronic acid, likely glucuronic acid. Q-10 is the predominant respiratory quinone, and the major polyamine is spermidine.

The type strain, *Novosphingobium oxfordense* BL-8A^*T*^ (NRRL B-65725^*T*^ = LMG 34032^*T*^), was isolated from the soil of a potted tomato plant grown in the city of Oxford, Mississippi, USA.

### 4.2 Description of *Novosphingobium mississippiense* sp. nov.

*Novosphingobium mississippiense* (mis.sis.sip.pi.en'se. N.L. neut. adj. *mississippiense*, pertaining to Mississippi, the state where the soil sample source of the type strain, a potted tomato plant, was grown).

Cells are Gram-stain-negative rods (1.1–1.4 μm). Colonies are yellow, circular and smooth after 48 h at 28 °C on LB. Grows at 4–40 °C (optimum, 28 °C), pH 4.0–10.5 (optimum, pH 7.0) and 0–7.5 % (w/v) NaCl supplementation to LB (optimum, 0–0.75 %). It contains a 6.1 Mb genome with four circular contigs, a G + C content of 64.4 mol %, and 5,723 CDS available on GenBank under the accession numbers CP147001–CP147004. Oxidase positive, while catalase and urease negative. It utilizes D-glucose, glycine, pyruvic acid, D-mannose, L-arabinose, D-galactose, D-glucuronic acid, maltose, *N*-acetyl glucosamine, citric acid and decanoic acid, it did not utilize D-mannitol as carbon source. The major ceramides are 528 *m/z*, 554 *m/z*, and 568 *m/z*, with each of these ceramides also being elaborated with a hexuronic acid, likely glucuronic acid. Q-10 is the predominant respiratory quinone, and the major polyamine is spermidine.

The type strain, *Novosphingobium mississippiense* BL-8H^*T*^ (NRRL B-65726^*T*^ = LMG 34033^*T*^), was isolated from the soil of a potted tomato plant grown in the city of Oxford, Mississippi, USA.

## Data Availability

MS data was deposited with the MassIVE archive and given the accession number MSV000095235. Preliminary 16S rRNA gene sequences were submitted to GenBank, accession numbers PQ559293 and PQ559294, as well as the near-complete 16S rRNA gene sequences, PX934875 and PX934876, for BL-8A^*T*^ and BL-8H^*T*^, respectively. The genomic data were also uploaded to GenBank under the BioProject number PRJNA1082396. Strains BL-8A^*T*^ and BL-8H^*T*^ have been deposited with the ATCC under the type strain deposit numbers TSD-464 and TSD-465 for BL-8A^*T*^ and BL-8H^*T*^, respectively, the NRRL under the collection numbers B-65725^*T*^ for BL-8A^*T*^ and B-65726^*T*^ for BL-8H^*T*^, and the BCCM/LMG under the collection numbers LMG 34032^*T*^ for BL-8A^*T*^ and LMG 34033^*T*^ for BL-8H^*T*^.
