# Supplementary material for: Isolation and characterization of Novosphingobium oxfordense sp. nov. and Novosphingobium mississippiense sp. nov. from soil, with LC-MS/MS and genome-based investigation of their glycosphingolipid production
Source: Front Microbiol. 2026 Jun 16;17:1862985. doi: 10.3389/fmicb.2026.1862985 (PMC13318209; doi:10.3389/fmicb.2026.1862985)
Supplement: Supplementary file 1 [file Data_Sheet_1.pdf]

Isolation and characterization of Novosphingobium oxfordense sp. nov. and Novosphingobium mississippiense sp. nov. from soil, with LC-MS/MS and genome-based investigation of their glycosphingolipid production

1 SUPPLEMENTARY TABLES AND FIGURES

1.1 Figures

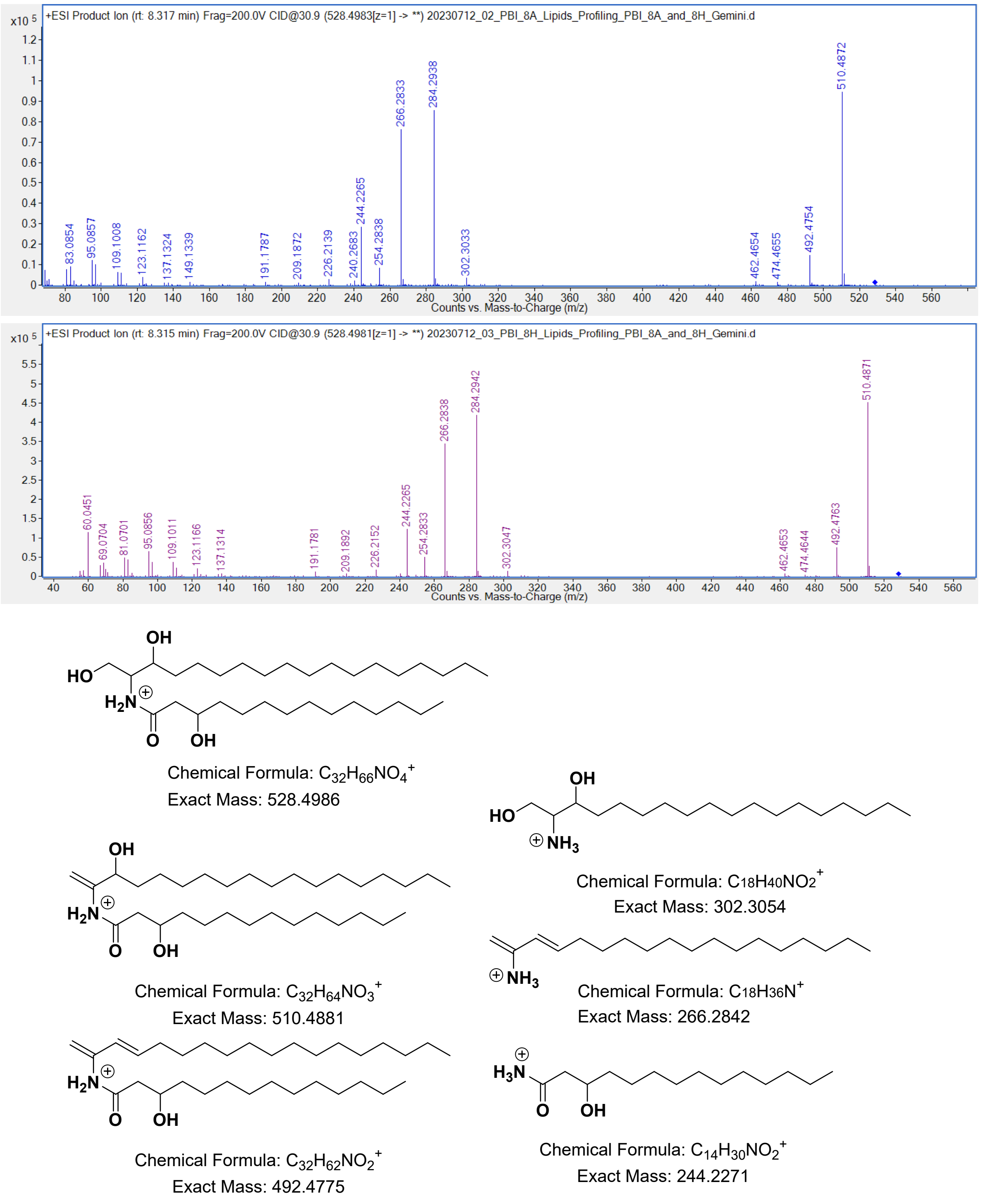

Figure S1: **HRMS^2 fragment spectrum of 528 *m/z*.** Spectra from BL-8A^T (top) and BL-8H^T (bottom), with annotated structures below.

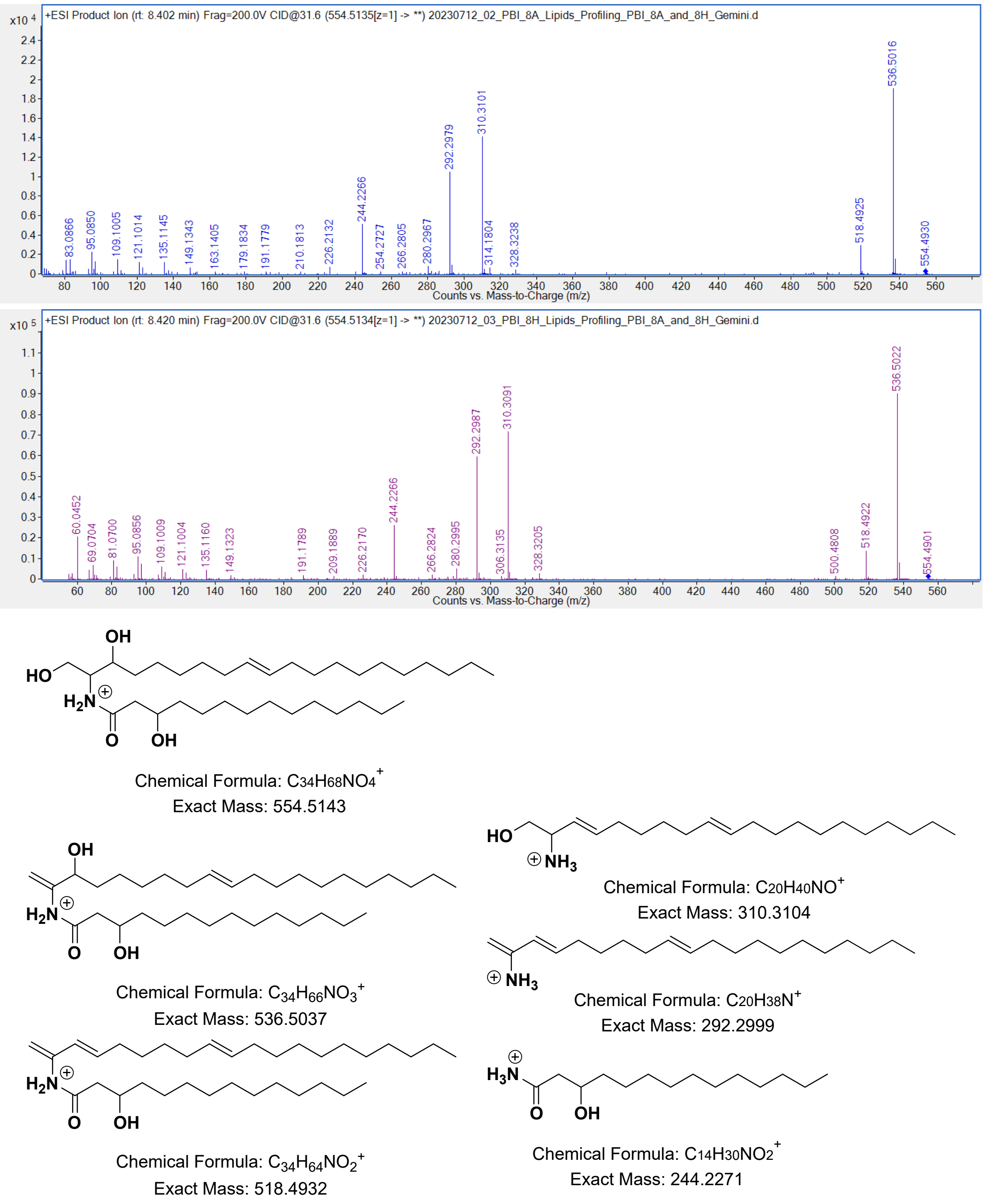

Figure S2: **HRMS^2 fragment spectrum of 554 *m/z*.** Spectra from BL-8A^T (top) and BL-8H^T (bottom), with annotated structures below. Note that the structures were derived from MS^2 fragment annotation, as such, the degree of unsaturation in the parent lipid is ambiguous. It could be a double bond or cyclopropane ring, and its placement is unknown.

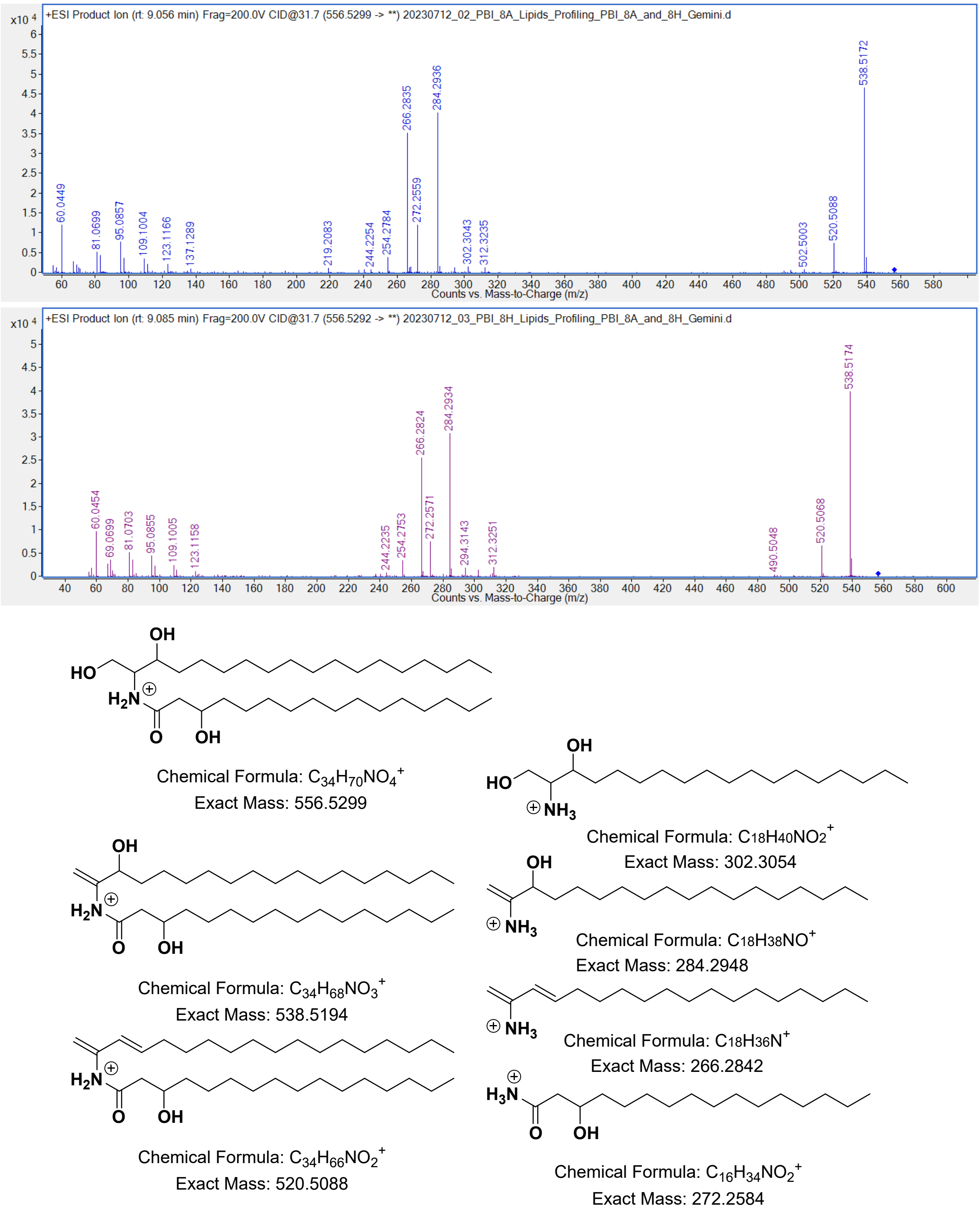

Figure S3: **HRMS^2 fragment spectrum of 556 *m/z*.** Spectra from BL-8A^T (top) and BL-8H^T (bottom), with annotated structures below.

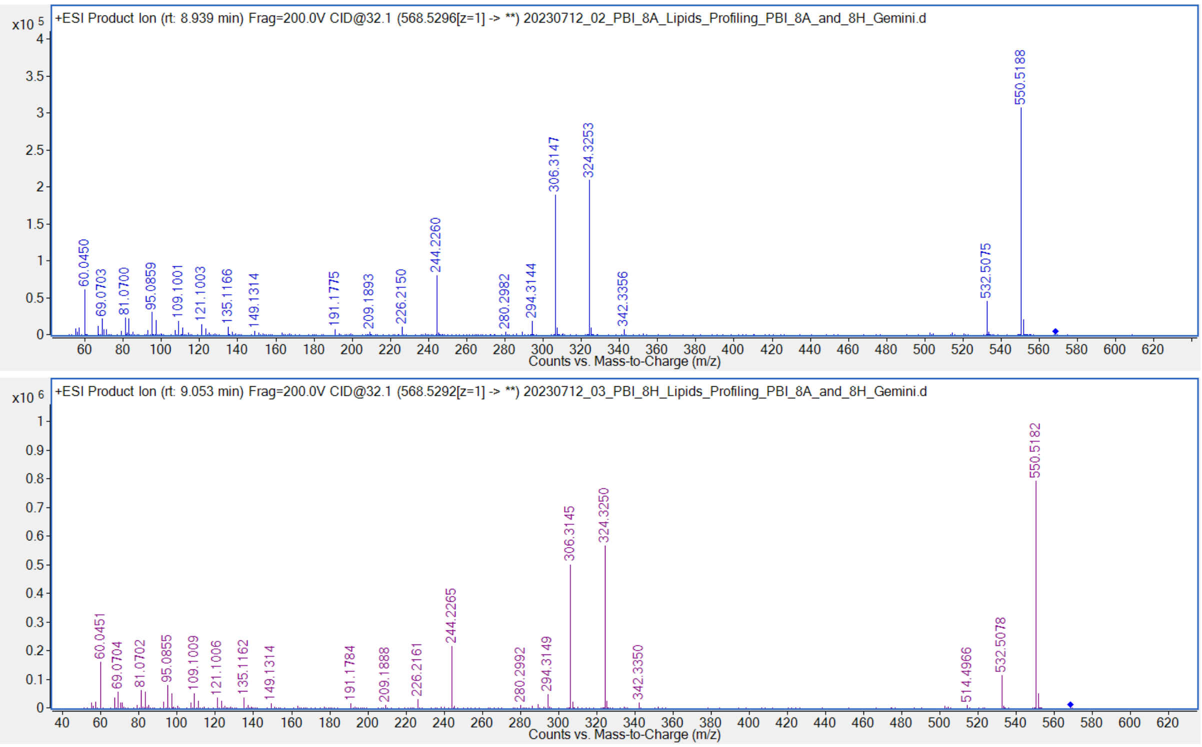

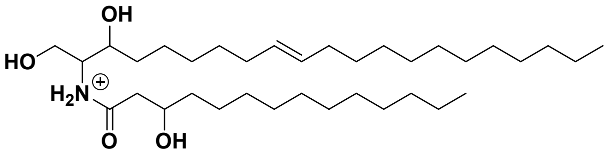

Chemical Formula: $C_{35}H_{70}NO_4^+$
Exact Mass: 568.5299

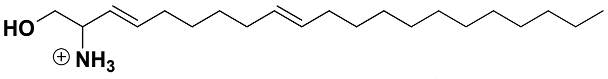

Chemical Formula: $C_{21}H_{42}NO^+$
Exact Mass: 324.3261

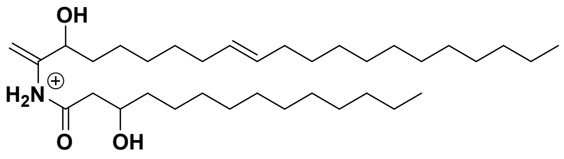

Chemical Formula: $C_{35}H_{68}NO_3^+$
Exact Mass: 550.5194

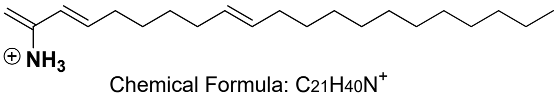

Chemical Formula: $C_{21}H_{40}N^+$
Exact Mass: 306.3155

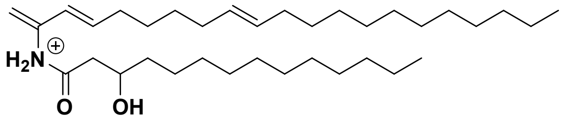

Chemical Formula: $C_{35}H_{66}NO_2^+$
Exact Mass: 532.5088

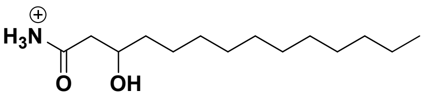

Chemical Formula: $C_{14}H_{30}NO_2^+$
Exact Mass: 244.2271

Figure S4: **HRMS^2 fragment spectrum of 568 *m/z*.** Spectra from BL-8A^T (top) and BL-8H^T (bottom), with annotated structures below. Note that the structures were derived from MS^2 fragment annotation, as such, the degree of unsaturation in the parent lipid is ambiguous. It could be a double bond or cyclopropane ring, and its placement is unknown.

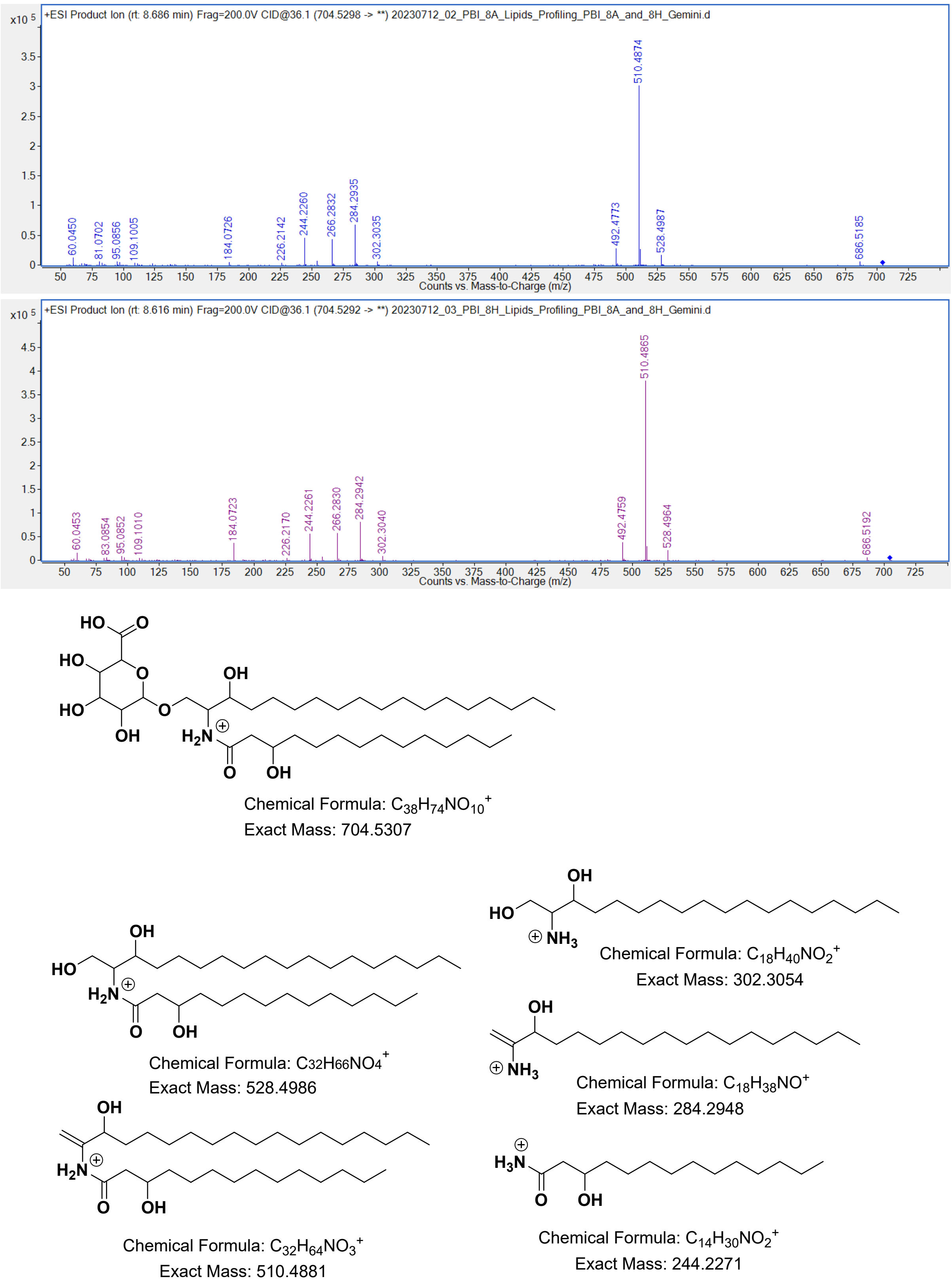

Figure S5: **HRMS^2 fragment spectrum of 704 *m/z*.** Spectra from BL-8A^T (top) and BL-8H^T (bottom), with annotated structures below.

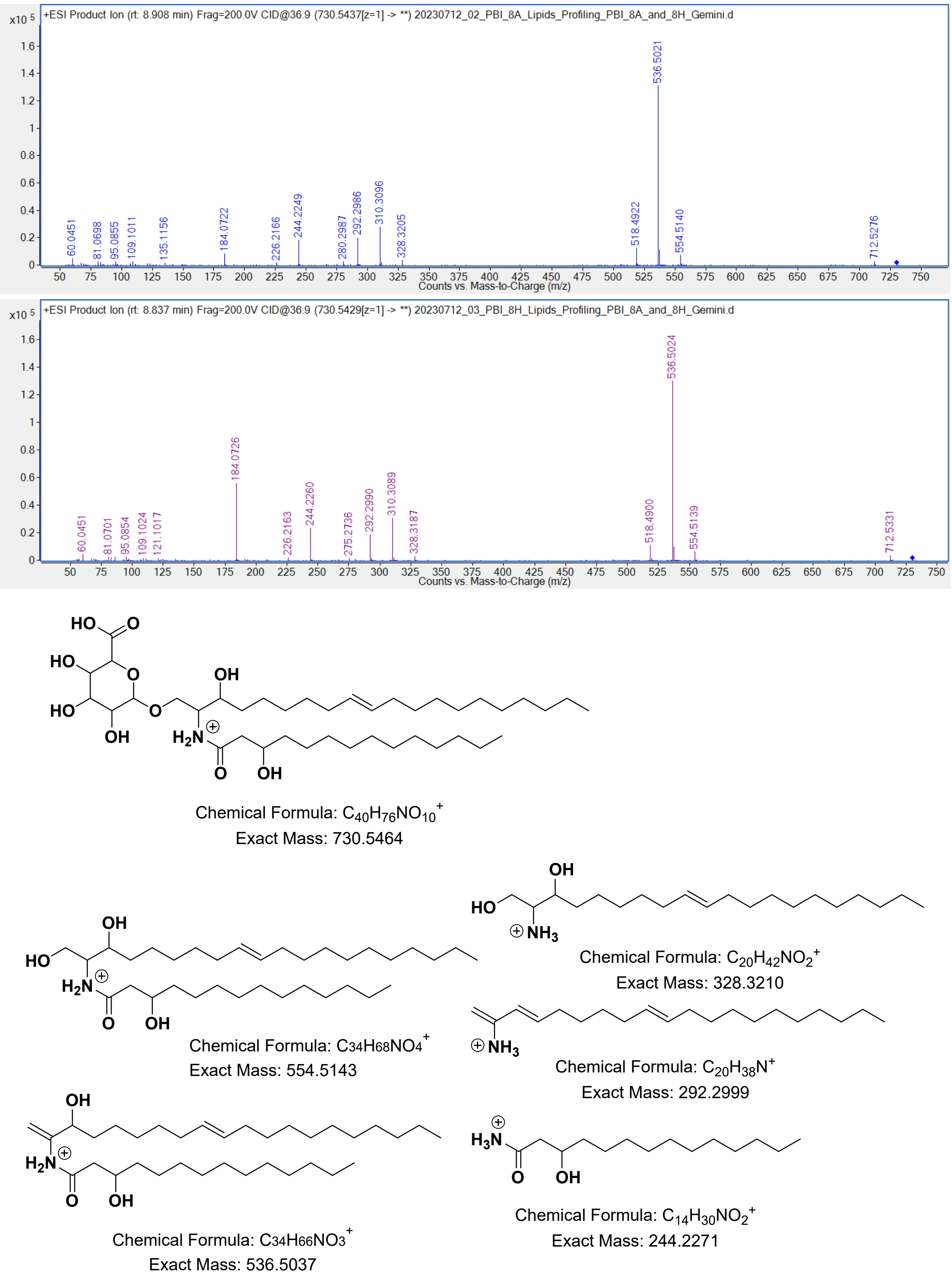

Figure S6: **HRMS^2 fragment spectrum of 730 *m/z*.** Spectra from BL-8A^T (top) and BL-8H^T (bottom), with annotated structures below. Note that the structures were derived from MS^2 fragment annotation, as such, the degree of unsaturation in the parent lipid is ambiguous. It could be a double bond or cyclopropane ring, and its placement is unknown.

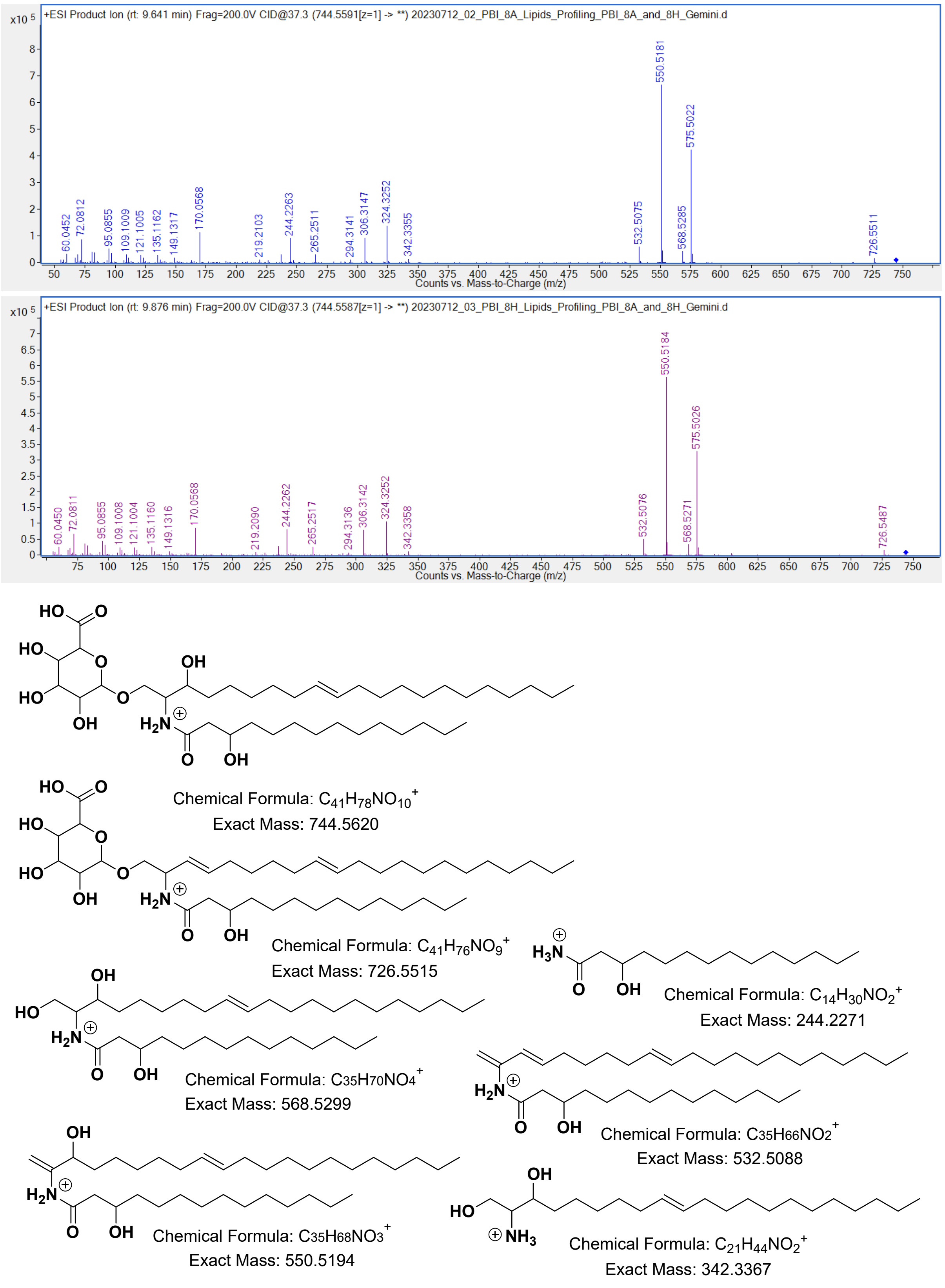

Figure S7: **HRMS^2 fragment spectrum of 744 *m/z*.** Spectra from BL-8A^T (top) and BL-8H^T (bottom), with annotated structures below. Note that the structures were derived from MS^2 fragment annotation, as such, the degree of unsaturation in the parent lipid is ambiguous. It could be a double bond or cyclopropane ring, and its placement is unknown.

	BL-8A	BL-8H
**Gram Staining**	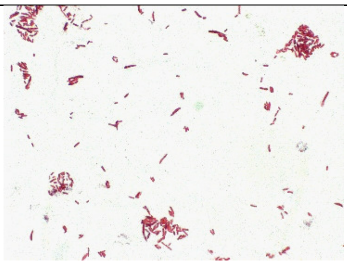	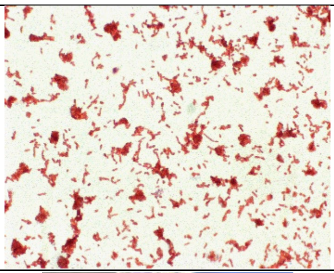
**Non-motile**	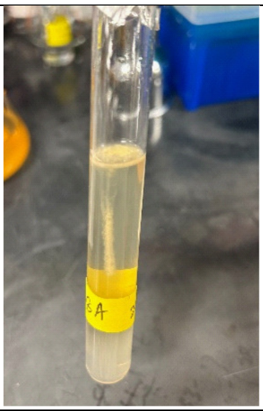	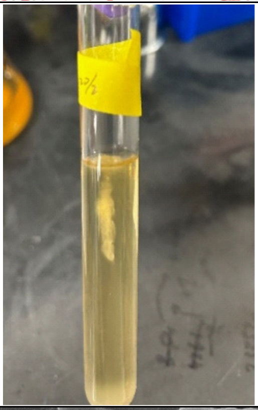
**Rod-shaped**	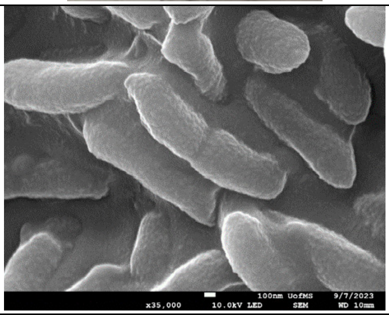	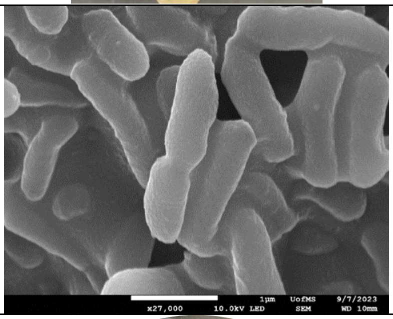
**Yellow colonies**	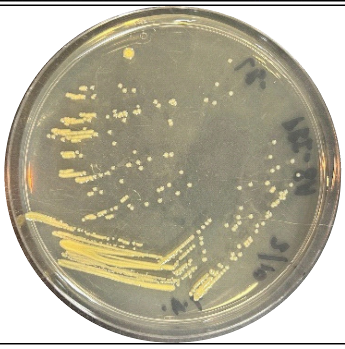	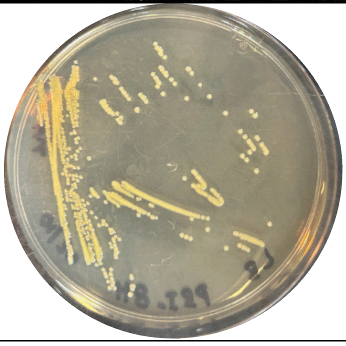

Figure S8: **Morphological features of strains** ***Novosphingobium oxfordense*** **BL-8A^{T} and** ***Novosphingobium mississippiense*** **BL-8H^{T}.**

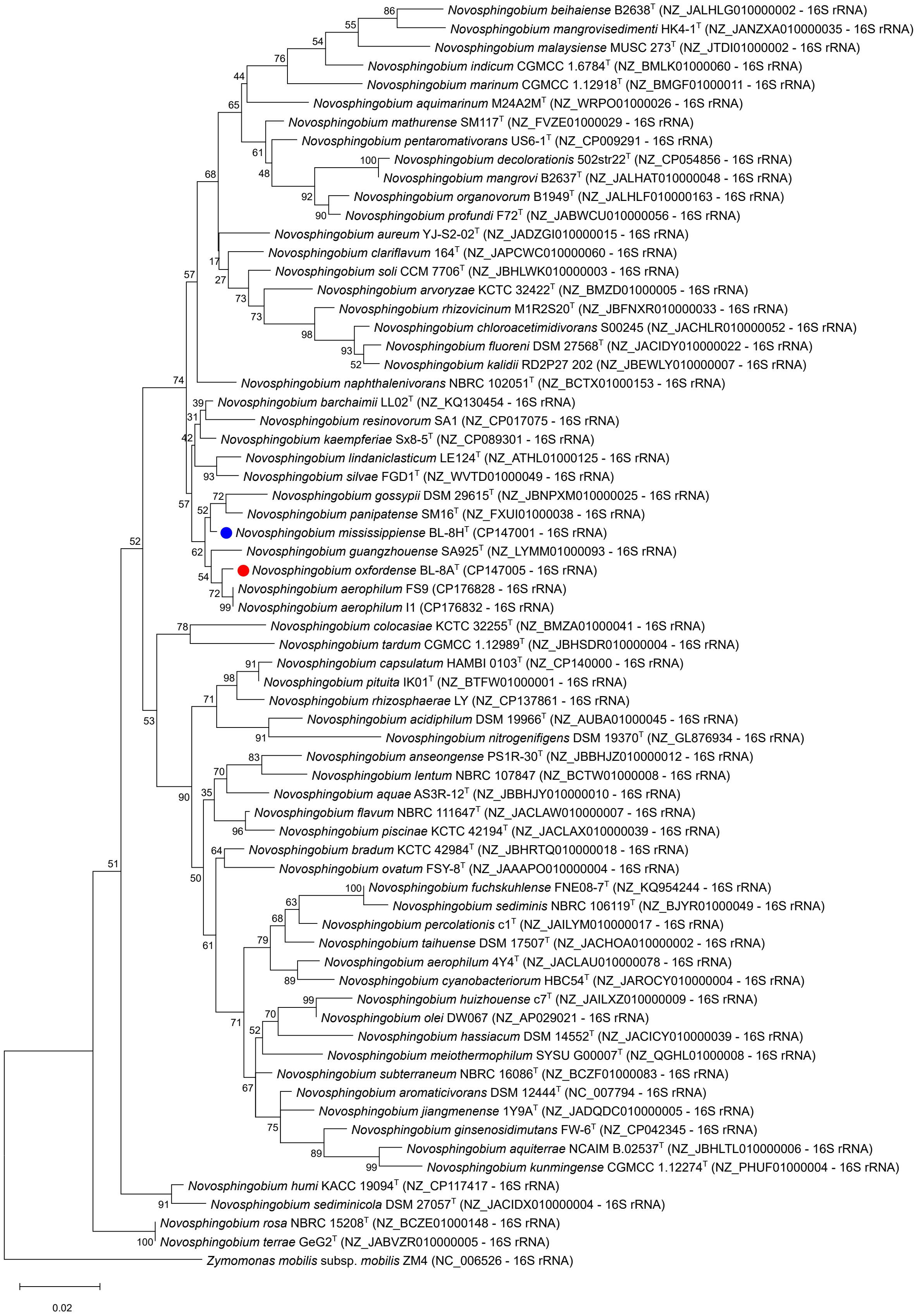

Figure S9: **16S rRNA gene phylogenic tree.** *(Caption on next page.)*

Figure S9: **16S rRNA gene phylogenic tree.** A Maximum Likelihood tree made with the Tamura-Nei model of nucleotide substitutions (Kumar et al., 2024; Tamura and Nei, 1993). For the figure the tree with the highest log likelihood (-11,065.48) was shown, rooted to the *Zymomonas* outgroup, with percentage of replicate trees in which the associated taxa clustered together out of 8,192 bootstraps also indicated at the branch point (Felsenstein, 1985). The initial tree for the heuristic search was selected by choosing the tree with the superior log-likelihood between a Neighbor-Joining tree (Saitou and Nei, 1987) and a Maximum Parsimony tree. The Neighbor-Joining tree was generated using a matrix of pairwise distances computed with the Tamura-Nei (1993) model (Tamura and Nei, 1993). The MP tree had the shortest length among 10 MP tree searches, each performed with a randomly generated starting tree. GenBank accession number from which the 16S rRNA gene was extracted indicated in the parenthetical.

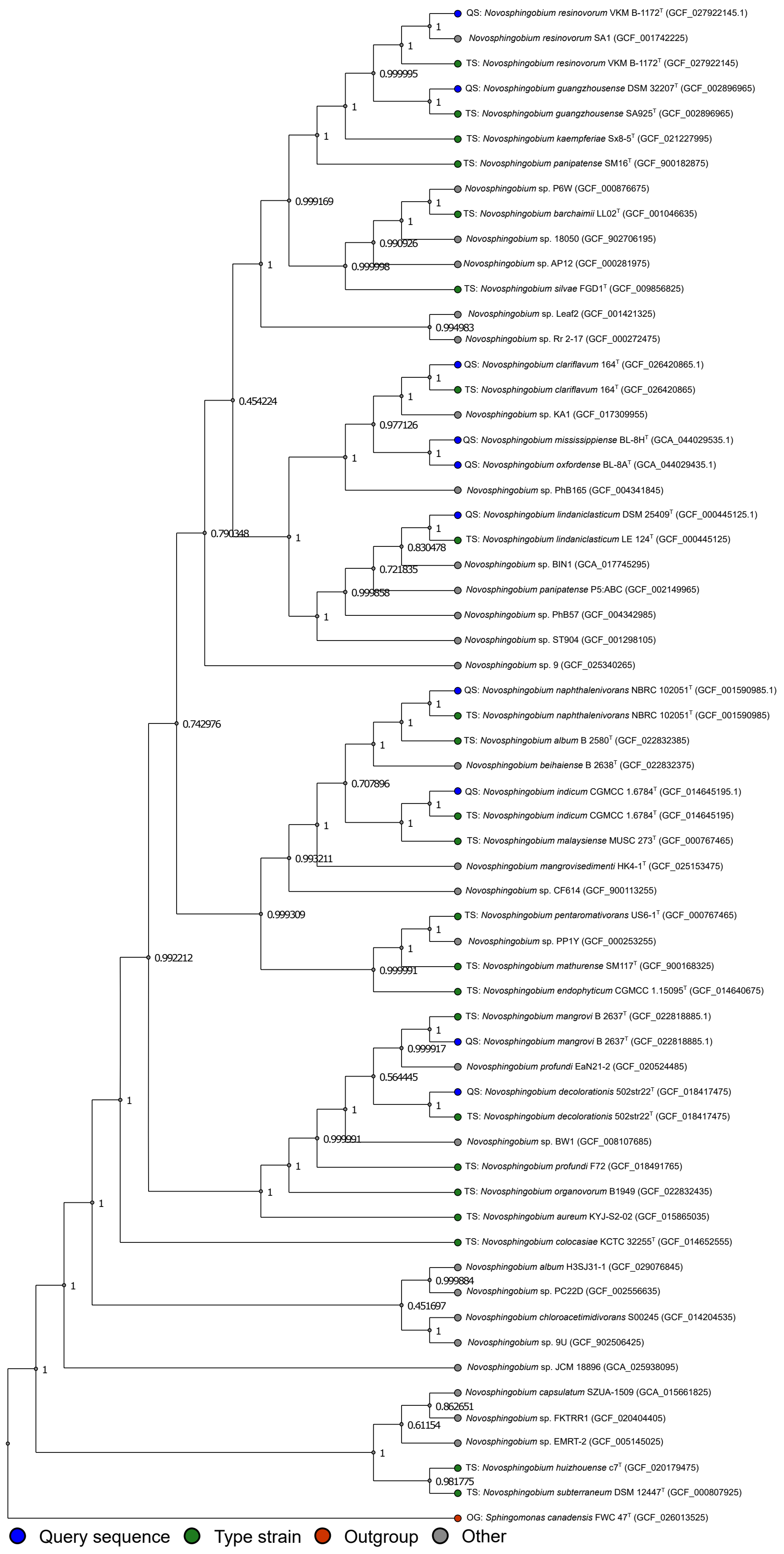

Figure S10: **autoMLST2 phylogenic tree.**The unscaled branches are shown with support indicated as proportions (0-1) at the branch point. GenBank genome accession number provided for each strain in the parenthetical.

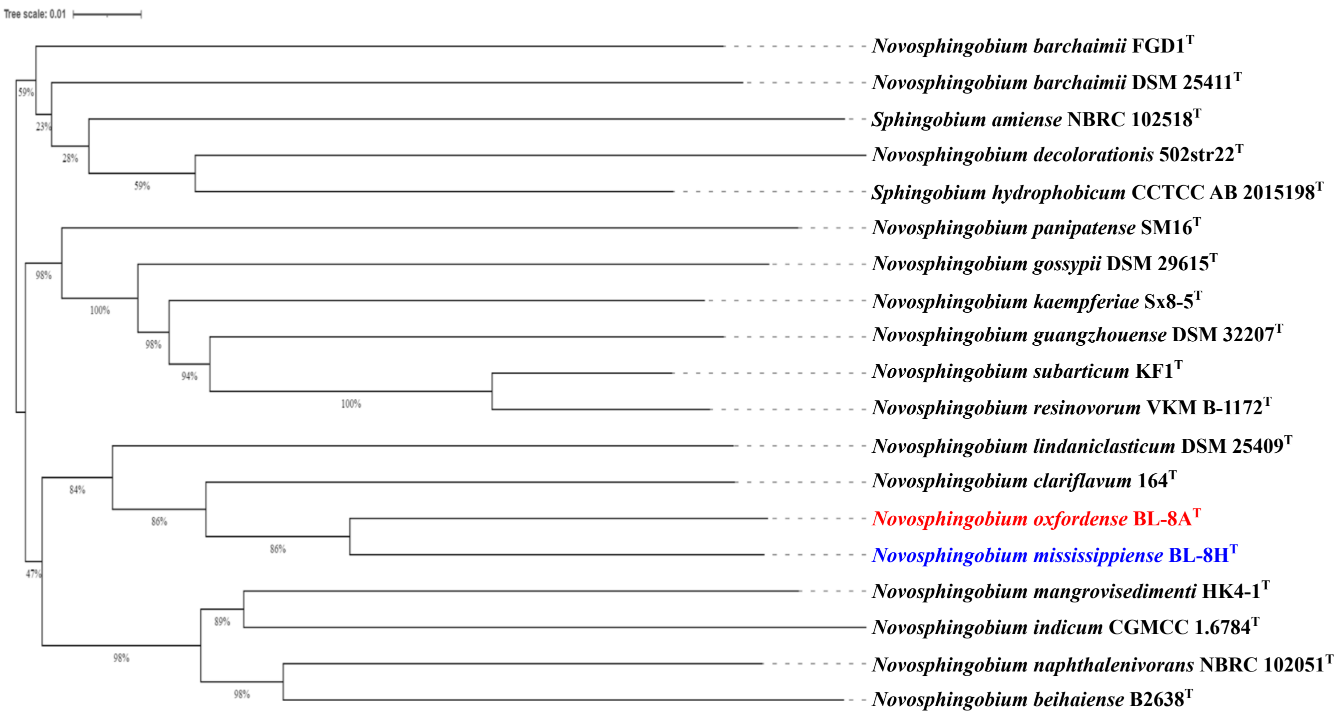

Figure S11: **Whole genome-based phylogenic tree.** Phylogenomic tree illustrating the relationship between strains BL-8A, BL-8H, and other closely related Novosphingobium strains. Constructed using the Genome Blast Distance Phylogeny (GBDP) approach and sourced from the TYGS website.

Table S1. Mass Annotations for the Sphingolipids.

Molecular Formula, Theoretical *m/z*	Observed HRMS/MS *m/z*	Error (ppm)
Parent mass and selected fragment ions for 528 *m/z*.		
$C_{32}H_{66}NO_4^+$, 528.4986	528.4983	-0.57
$C_{32}H_{64}NO_3^+$, 510.4881	510.4872	-1.76
$C_{32}H_{62}NO_2^+$, 492.4775	492.4754	-4.26
$C_{18}H_{40}NO_2^+$, 302.3054	302.3033	-6.94
$C_{18}H_{36}N^+$, 266.2842	266.2833	-3.37
$C_{14}H_{30}NO_2^+$, 244.2271	244.2265	-2.45
Parent mass and selected fragment ions for 554 *m/z*.		
$C_{34}H_{68}NO_4^+$, 554.5143	554.5135	-1.44
$C_{34}H_{66}NO_3^+$, 536.5037	536.5016	-3.91
$C_{34}H_{64}NO_2^+$, 518.4932	518.4925	-1.35
$C_{20}H_{40}NO^+$, 310.3104	310.3101	-0.96
$C_{20}H_{38}N^+$, 292.2999	292.2979	-6.84
$C_{14}H_{30}NO_2^+$, 244.2271	244.2266	-2.05
Parent mass and selected fragment ions for 556 *m/z*.		
$C_{34}H_{70}NO_4^+$, 556.5299	556.5299	0
$C_{34}H_{68}NO_3^+$, 538.5194	538.5174	-3.71
$C_{34}H_{66}NO_2^+$, 520.5088	520.5068	-3.84
$C_{18}H_{40}NO_2^+$, 302.3054	302.3043	-3.63
$C_{18}H_{38}NO^+$, 284.2948	284.2936	-4.22
$C_{16}H_{34}NO_2^+$, 272.2584	272.2571	-4.77
$C_{18}H_{36}N^+$, 266.2842	266.2835	-2.63
Parent mass and selected fragment ions for 568 *m/z*.		
$C_{35}H_{70}NO_4^+$, 568.5299	568.5296	-0.51
$C_{35}H_{68}NO_3^+$, 550.5194	550.5188	-1.08
$C_{35}H_{66}NO_2^+$, 532.5088	532.5075	-2.44
$C_{21}H_{42}NO^+$, 324.3261	324.3253	-2.46
$C_{21}H_{40}N^+$, 306.3155	306.3147	-2.61
$C_{14}H_{30}NO_2^+$, 244.2271	244.2260	-4.50
Parent mass and selected fragment ions for 704 *m/z*.		
$C_{38}H_{74}NO_{10}^+$, 704.5307	704.5298	-1.27
$C_{32}H_{66}NO_4^+$, 528.4986	528.4987	0.19
$C_{32}H_{64}NO_3$, 510.4881	510.4874	-1.37
$C_{18}H_{40}NO_2^+$, 302.3054	302.3035	-6.28
$C_{18}H_{38}NO^+$, 284.2948	284.2935	-4.57
$C_{14}H_{30}NO_2^+$, 244.2271	244.2260	-4.50
Parent mass and selected fragment ions for 730 *m/z*.		
$C_{40}H_{76}NO_{10}^+$, 730.5464	730.5437	-3.69
$C_{34}H_{68}NO_4^+$, 554.5143	554.5140	-0.54
$C_{34}H_{66}NO_3^+$, 536.5037	536.5021	-2.98
$C_{20}H_{42}NO_2^+$, 328.3210	328.3205	-1.52

Continued on next page

**Molecular Formula, Theoretical *m/z***	**Observed HRMS/MS *m/z***	**Error (ppm)**
Parent mass and selected fragment ions for 730 *m/z*. *(continued)*		
$C_{20}H_{38}N^{+}$, 292.2999	292.2986	-4.45
$C_{14}H_{30}NO_2^{+}$, 244.2271	244.2249	-9.01
Parent mass and selected fragment ions for 744 *m/z*.		
$C_{41}H_{78}NO_{10}^{+}$, 744.5620	744.5591	-3.89
$C_{41}H_{76}NO_9^{+}$, 726.5515	726.5511	-0.55
$C_{35}H_{70}NO_4^{+}$, 568.5299	568.5285	-2.46
$C_{35}H_{68}NO_3^{+}$, 550.5194	550.5181	-2.36
$C_{35}H_{66}NO_2^{+}$, 532.5088	532.5075	-2.44
$C_{21}H_{42}NO^{+}$, 324.3261	324.3252	-2.77
$C_{14}H_{30}NO_2^{+}$, 244.2271	244.2263	-3.28

Table S2. Complete Physiological and Biochemical Differences of Strains BL-8A^T and BL-8H^T Compared to Representatives of the Genus.

Continued from **Table 1** in the main text.
Strains: **1** = *N. oxfordense* BL-8A^T, **2** = *N. mississippiense* BL-8H^T,
3 = *N. clariflavum* 164^T (DSM 103351), **4** = *N. guangzhouense* DSM 32207^T.
+ = Positive, - = Negative, w = Weakly positive

**Characteristics**	**1**	**2**	**3**	**4**
Isolation Source	Soil	Soil	Sponge	Soil
DNA G+C content (mol%)	64.0	64.4	65.9	60.2
Motility	-	-	-	-
Optimal temperature for growth (°C)	28	28	28	28
pH range for growth:				
in buffered TSB	5.0–7.5	5.0–8.0	5.0–8.0	6.0–8.0
in buffered LB	5.0–7.5	5.0–8.0	5.0–7.0	5.0–8.0
Supplemental NaCl (%) range for growth	0–3.0	0–3.0	0–2.0	0–4.0
Growth on LB	+	+	+	+
Growth on DMS	+	+	+	+
Growth on TSA	+	+	+	+
Nitrate reduction	-	+	-	-
Oxidase Activity	+	+	-	+
Catalase Activity	+	-	-	-
Hydrolysis of:				
Tween 40	-	-	-	w
Tween 80	-	-	-	-
Starch	-	+	-	+
Casein	-	-	-	-
Enzyme Assays				
Glucose utilization	-	-	-	-
Arginine Dihydrolase	-	-	-	-
Hydrolysis (β-Glucosidase) Esculin	+	+	+	+
Hydrolysis (protease) Gelatin	-	-	-	-
β-Galactosidase	+	+	+	+
Assimilation of D-Glucose	+	+	+	+
Assimilation of L-Arabinose	+	+	+	+
Assimilation of D-Mannose	+	+	+	+
Assimilation of D-Mannitol	-	-	-	-
Assimilation of *N*-Acetyl-Glucosamine	+	+	-	+
Assimilation of D-Maltose	+	+	+	+
Assimilation of Potassium Gluconate	+	+	-	-
Assimilation of Capric Acid	w	-	-	-
Assimilation of Adipic Acid	w	w	-	-
Assimilation of Malic Acid	-	w	+	-
Assimilation of Trisodium Citrate	-	-	-	+

Continued on next page

(Continued from previous page)

**Characteristics**	**1**	**2**	**3**	**4**
Assimilation of Phenylacetic Acid	-	-	-	-
Carbon source utilization:				
L-Arabinose	+	+	+	+
D-Glucose	+	+	+	+
D-Galactose	+	+	+	+
D-Mannitol	-	-	+	+
D-Mannose	+	+	+	+
Pyruvic acid	+	+	+	+
Citric acid	+	+	+	+
D-Glucuronic acid	+	+	+	+
Maltose	+	+	+	+
N-Acetyl-glucosamine	+	+	+	+
Decanoic acid	+	+	+	+
Antibiotic resistance:				
Ampicillin	-	-	-	-
Ciprofloxacin	-	-	-	-
Chloramphenicol	-	+	+	+
Carbenicillin	-	-	-	-
Kanamycin	-	-	-	-
Streptomycin	+	+	+	+
Tetracycline	-	-	-	-

Table S3. Digital DNA-DNA Hybridization(dDDH) Comparisons vs. Type-Strain Genomes via TYGS.

Query strain	Subject strain	dDDH (d4, %)
N. oxfordense BL-8A^T	*N. mississippiense* BL-8H^T	34.8
N. oxfordense BL-8A^T	*Sphingobium hydrophobicum* CCTCC AB 2015198^T	30.3
N. mississippiense BL-8H^T	*Novosphingobium clariflavum* 164^T	28.3
N. oxfordense BL-8A^T	*Novosphingobium clariflavum* 164^T	28.2
N. oxfordense BL-8A^T	*Sphingobium amiense* NBRC 102518^T	26.5
N. mississippiense BL-8H^T	*Novosphingobium lindaniclasticum* DSM 25409^T	25.0
N. oxfordense BL-8A^T	*Novosphingobium lindaniclasticum* DSM 25409^T	24.7
N. oxfordense BL-8A^T	*Novosphingobium subarcticum* KF1^T	24.1
N. mississippiense BL-8H^T	*Novosphingobium guangzhouense* DSM 32207^T	22.9
N. mississippiense BL-8H^T	*Novosphingobium resinovorum* VKM B-1172^T	22.6
N. mississippiense BL-8H^T	*Novosphingobium panipatense* SM16^T	22.5
N. mississippiense BL-8H^T	*Novosphingobium kaempferiae* Sx8-5^T	22.4
N. mississippiense BL-8H^T	*Novosphingobium subarcticum* KF1^T	22.3
N. oxfordense BL-8A^T	*Novosphingobium resinovorum* VKM B-1172^T	22.2
N. oxfordense BL-8A^T	*Novosphingobium kaempferiae* Sx8-5^T	22.2
N. mississippiense BL-8H^T	*Novosphingobium naphthalenivorans* NBRC 102051^T	22.1
N. oxfordense BL-8A^T	*Novosphingobium naphthalenivorans* NBRC 102051^T	22.0
N. mississippiense BL-8H^T	*Novosphingobium decolorationis* 502str22^T	21.9
N. mississippiense BL-8H^T	*Novosphingobium gossypii* DSM 29615^T	21.8
N. oxfordense BL-8A^T	*Novosphingobium gossypii* DSM 29615^T	21.8
N. mississippiense BL-8H^T	*Novosphingobium barchaimii* DSM 25411^T	21.8
N. oxfordense BL-8A^T	*Novosphingobium barchaimii* DSM 25411^T	21.7
N. oxfordense BL-8A^T	*Novosphingobium mangrovi* HK4-1^T	21.7
N. oxfordense BL-8A^T	*Novosphingobium beihaiensis* B2638^T	21.7
N. mississippiense BL-8H^T	*Novosphingobium mangrovi* HK4-1^T	21.7
N. mississippiense BL-8H^T	*Novosphingobium beihaiensis* B2638^T	21.7
N. oxfordense BL-8A^T	*Novosphingobium guangzhouense* DSM 32207^T	21.6
N. oxfordense BL-8A^T	*Novosphingobium silvae* FGD1^T	21.5
N. oxfordense BL-8A^T	*Novosphingobium panipatense* SM16^T	21.4
N. mississippiense BL-8H^T	*Novosphingobium silvae* FGD1^T	21.4
N. oxfordense BL-8A^T	*Novosphingobium indicum* CGMCC 1.6784^T	21.4
N. mississippiense BL-8H^T	*Novosphingobium indicum* CGMCC 1.6784^T	21.3
N. oxfordense BL-8A^T	*Novosphingobium decolorationis* 502str22^T	21.0
N. mississippiense BL-8H^T	*Sphingobium hydrophobicum* CCTCC AB 2015198^T	19.9
N. mississippiense BL-8H^T	*Sphingobium amiense* NBRC 102518^T	19.6

Table S4. Complete orthoANI Analysis of *Novosphingobium oxfordense* BL-8A^T (Lee et al., 2016).

Accession Code	OrthoANI (%)	Accession Code	OrthoANI (%)
GCA_044029435.1	100	GCA_044029535.1	87.5477
GCA_943913065.1	83.7055	GCA_042434475.1	83.6959
GCA_026420865.1	83.5639	GCA_017309955.1	83.4035
GCA_004341845.1	83.3963	GCA_032099065.1	81.3251
GCA_032017565.1	81.2606	GCA_003241455.1	81.2385
GCA_009811895.1	81.1366	GCA_050725775.1	80.9072
GCA_017745295.1	80.8911	GCA_963522375.1	80.8564
GCA_009360525.1	80.813	GCA_004342985.1	80.7681
GCA_004368805.1	80.7428	GCA_032054785.1	80.6147
GCA_036820275.1	80.567	GCA_002149965.1	80.5667
GCA_902168295.1	80.5003	GCA_044987305.1	80.393
GCA_035658495.1	80.2823	GCA_032093395.1	80.1871
GCA_004341305.1	79.985	GCA_032104605.1	79.9704
GCA_001298105.1	79.8936	GCA_029239695.1	79.8787
GCA_000445125.1	79.7246	GCA_000632105.1	78.6764
GCA_022832375.1	77.8624	GCA_025153475.1	77.8528
GCA_042682425.1	77.8297	GCA_001590985.1	77.7674
GCA_035511275.1	77.7301	GCA_027922145.1	77.7048
GCA_030209485.1	77.6585	GCA_036352595.1	77.65
GCA_042432915.1	77.6272	GCA_042660805.1	77.6189
GCA_050470805.1	77.6154	GCA_041276605.1	77.606
GCA_021227995.1	77.5893	GCA_042683865.1	77.5786
GCA_000281975.1	77.4077	GCA_001742225.1	77.4071
GCA_900113255.1	77.3606	GCA_044813085.1	77.3102
GCA_037384205.1	77.2148	GCA_015594425.1	77.1934
GCA_965140495.1	77.1241	GCA_037384285.1	77.0958
GCA_030369695.1	77.0764	GCA_022832385.1	77.0697
GCA_902706195.1	77.0459	GCA_902706245.1	76.9767
GCA_050433305.1	76.9737	GCA_001046635.1	76.9436
GCA_035278205.1	76.9425	GCA_001368935.1	76.8508
GCA_000253255.1	76.8155	GCA_047605225.1	76.8053
GCA_002896965.1	76.8024	GCA_014645195.1	76.7491
GCA_000767465.1	76.7441	GCA_009856825.1	76.7284
GCA_017312445.1	76.7249	GCA_900168325.1	76.6968
GCA_000876675.2	76.6507	GCA_014640675.1	76.613
GCA_000802225.1	76.5902	GCA_000235975.2	76.5679
GCA_044340705.1	76.5253	GCA_022832435.1	76.5227
GCA_042683985.1	76.5118	GCA_014652555.1	76.4476
GCA_050251905.1	76.4064	GCA_029076845.1	76.3944
GCA_001421325.1	76.3547	GCA_041911635.1	76.2317
GCA_015865035.1	76.2277	GCA_002684595.1	76.2216

Continued on next page

(Continued from previous page)

Accession Code	OrthoANI (%)	Accession Code	OrthoANI (%)
GCA_000272475.1	76.2167	GCA_025340265.1	76.1938
GCA_035276965.1	76.0351	GCA_018491765.1	75.8742
GCA_002916655.1	75.827	GCA_042661245.1	75.8228
GCA_042646585.1	75.8165	GCA_047246705.1	75.7792
GCA_042681875.1	75.7587	GCA_900182875.1	75.7336
GCA_018417475.1	75.7185	GCA_036733805.1	75.6577
GCA_014204535.1	75.6145	GCA_022818885.1	75.592
GCA_020524485.1	75.5266	GCA_000813185.1	75.4132
GCA_902506425.1	75.3427	GCA_913775085.1	75.2352
GCA_008107685.1	75.2035	GCA_036384395.1	75.1955
GCA_041677785.1	75.1817	GCA_913776895.1	75.1182
GCA_002556635.1	75.1152	GCA_913777595.1	75.0771
GCA_913778265.1	75.0584	GCA_964502105.1	74.9772
GCA_913777775.1	74.9464	GCA_913774105.1	74.9313
GCA_913776985.1	74.8939	GCA_913778915.1	74.8925
GCA_913776335.1	74.8395	GCA_913777055.1	74.8243
GCA_913774745.1	74.7835	GCA_001476695.1	74.7463
GCA_964277725.1	74.724	GCA_020848655.1	74.6354
GCA_014196615.1	74.6301	GCA_031285165.1	74.626
GCA_913775225.1	74.6236	GCA_913778115.1	74.6225
GCA_913776345.1	74.5585	GCA_964277855.1	74.5433
GCA_913775135.1	74.5181	GCA_913775125.1	74.5063
GCA_041912915.1	74.4598	GCA_047605105.1	74.4469
GCA_913777965.1	74.43	GCA_902168285.1	74.4211
GCA_913777865.1	74.3998	GCA_009746585.1	74.3977
GCA_964277795.1	74.3933	GCA_964520765.1	74.3879
GCA_014652615.1	74.3827	GCA_042646485.1	74.3594
GCA_913777155.1	74.3337	GCA_041909515.1	74.3161
GCA_913778675.1	74.3072	GCA_002855555.1	74.2637
GCA_025938095.1	74.2554	GCA_027532205.1	74.233
GCA_021299075.1	74.2261	GCA_002279725.1	74.2258
GCA_041912655.1	74.2243	GCA_004211435.1	74.2161
GCA_963923805.1	74.2062	GCA_027531025.1	74.2051
GCA_042434435.1	74.2018	GCA_027532385.1	74.1894
GCA_027532715.1	74.1704	GCA_046307415.1	74.1704
GCA_027530785.1	74.163	GCA_027530385.1	74.1518
GCA_964619635.1	74.1458	GCA_913775435.1	74.1442
GCA_027531865.1	74.1441	GCA_037482895.1	74.1276
GCA_041919975.1	74.1086	GCA_035430905.1	74.1048
GCA_027531065.1	74.0987	GCA_913778475.1	74.0939
GCA_938005655.1	74.083	GCA_018434425.1	74.0756
GCA_035281095.1	74.0624	GCA_028697875.1	74.0564

Continued on next page

(Continued from previous page)

Accession Code	OrthoANI (%)	Accession Code	OrthoANI (%)
GCA_041905565.1	74.0531	GCA_014230315.1	74.0513
GCA_014230305.1	74.05	GCA_913778485.1	74.0478
GCA_042649005.1	74.0469	GCA_035350035.1	74.0458
GCA_016719755.1	74.0286	GCA_046300735.1	74.0134
GCA_041915465.1	74.0009	GCA_035413165.1	73.9995
GCA_964561375.1	73.9933	GCA_964533975.1	73.9874
GCA_046307075.1	73.9798	GCA_014230355.1	73.9773
GCA_014230345.1	73.9746	GCA_016702815.1	73.9732
GCA_024639725.1	73.9696	GCA_046302515.1	73.9482
GCA_050281025.1	73.9361	GCA_025938155.1	73.9352
GCA_019075875.1	73.8794	GCA_937864195.1	73.8776
GCA_020179425.1	73.8671	GCA_027489625.1	73.8537
GCA_001014975.1	73.8534	GCA_046339575.1	73.8529
GCA_035423205.1	73.8434	GCA_035324535.1	73.8404
GCA_020179475.1	73.8384	GCA_030699305.1	73.8316
GCA_035386545.1	73.8266	GCA_937873015.1	73.8263
GCA_015661825.1	73.826	GCA_937996535.1	73.8224
GCA_040534015.1	73.8208	GCA_039927935.1	73.8143
GCA_964627175.1	73.7945	GCA_005145025.1	73.7871
GCA_039917735.1	73.7773	GCA_035327785.1	73.7741
GCA_015694345.1	73.7493	GCA_041424155.1	73.732
GCA_964413885.1	73.7284	GCA_015390225.1	73.7123
GCA_035416145.1	73.7095	GCA_046074165.1	73.6908
GCA_033242265.1	73.6901	GCA_024236175.1	73.6789
GCA_040813635.1	73.6678	GCA_050725835.1	73.6505
GCA_003856955.1	73.6405	GCA_030182715.1	73.6271
GCA_964573075.1	73.6164	GCA_040377095.1	73.6096
GCA_938001925.1	73.6054	GCA_002336885.1	73.6007
GCA_002279875.1	73.5983	GCA_015657645.1	73.5918
GCA_039914785.1	73.5889	GCA_002454125.1	73.571
GCA_036400975.1	73.5696	GCA_037076535.1	73.5619
GCA_046340395.1	73.5593	GCA_046052725.1	73.5572
GCA_039915025.1	73.5484	GCA_023228625.1	73.542
GCA_031421295.1	73.534	GCA_035543295.1	73.493
GCA_046306915.1	73.4819	GCA_001725355.1	73.4777
GCA_964621355.1	73.4685	GCA_000013325.1	73.467
GCA_041396785.1	73.4507	GCA_024236215.1	73.4371
GCA_013822565.1	73.4316	GCA_031422455.1	73.4314
GCA_964343385.1	73.4271	GCA_013408095.1	73.4263
GCA_038921155.1	73.4155	GCA_032337075.1	73.4089
GCA_046063245.1	73.407	GCA_035328685.1	73.3988
GCA_036383795.1	73.3978	GCA_027325615.1	73.3822

Continued on next page

(Continued from previous page)

Accession Code	OrthoANI (%)	Accession Code	OrthoANI (%)
GCA_964493835.1	73.3722	GCA_025938935.1	73.3708
GCA_041910515.1	73.3693	GCA_012927405.1	73.3664
GCA_046291335.1	73.3617	GCA_029436685.1	73.3577
GCA_035336345.1	73.348	GCA_040079495.1	73.3291
GCA_035345455.1	73.3188	GCA_046294595.1	73.3172
GCA_039543125.1	73.3144	GCA_007954425.1	73.3119
GCA_019510335.1	73.3108	GCA_040007615.1	73.3077
GCA_900102455.1	73.2931	GCA_000410615.1	73.2883
GCA_020852455.1	73.2862	GCA_014652855.1	73.2766
GCA_046291115.1	73.2761	GCA_003171715.1	73.2503
GCA_046063225.1	73.2492	GCA_046307775.1	73.2469
GCA_036803765.1	73.2395	GCA_043785715.1	73.2348
GCA_036784765.1	73.2344	GCA_003454795.1	73.2343
GCA_002813245.1	73.2302	GCA_046290865.1	73.2224
GCA_042685765.1	73.2141	GCA_031428115.1	73.2017
GCA_037572715.1	73.1949	GCA_964574135.1	73.1895
GCA_035426945.1	73.188	GCA_028292865.1	73.1829
GCA_014640055.1	73.181	GCA_042648825.1	73.181
GCA_041909635.1	73.166	GCA_041915005.1	73.1627
GCA_013141325.1	73.1604	GCA_964573265.1	73.1463
GCA_007830315.1	73.1273	GCA_003050985.1	73.1236
GCA_003058045.1	73.1236	GCA_003058145.1	73.1188
GCA_003058095.1	73.1187	GCA_964242815.1	73.118
GCA_046291445.1	73.1135	GCA_035563755.1	73.1028
GCA_036275395.1	73.0927	GCA_014193715.1	73.0883
GCA_964468165.1	73.0611	GCA_014194895.1	73.0594
GCA_019748305.1	73.0554	GCA_945906005.1	73.0533
GCA_000807925.1	73.0525	GCA_031368085.1	73.0504
GCA_035571815.1	73.0472	GCA_035719485.1	73.0471
GCA_014195215.1	73.046	GCA_043734515.1	73.0423
GCA_027311705.1	73.0416	GCA_020200285.1	73.0413
GCA_012641315.1	73.036	GCA_020446565.1	73.0357
GCA_001295765.1	73.0356	GCA_014193455.1	73.032
GCA_027533085.1	73.0288	GCA_001598575.1	73.0262
GCA_014195075.1	73.0241	GCA_014193775.1	73.0232
GCA_014193535.1	73.0204	GCA_031360905.1	73.0204
GCA_015169775.1	73.0179	GCA_020446485.1	73.0074
GCA_042662505.1	73.0061	GCA_027340905.1	72.9988
GCA_035653935.1	72.9956	GCA_046070605.1	72.9893
GCA_050920675.1	72.9844	GCA_002198665.1	72.9834
GCA_046063265.1	72.983	GCA_002279815.1	72.9774
GCA_031454595.1	72.9743	GCA_002440635.1	72.9733

Continued on next page

(Continued from previous page)

Accession Code	OrthoANI (%)	Accession Code	OrthoANI (%)
GCA_040544855.1	72.9706	GCA_014193475.1	72.9637
GCA_044378785.1	72.9615	GCA_013822005.1	72.9595
GCA_031364005.1	72.9573	GCA_038921205.1	72.956
GCA_964563565.1	72.9524	GCA_001590965.1	72.9487
GCA_041396825.1	72.9452	GCA_014193835.1	72.9406
GCA_050281045.1	72.9393	GCA_964550775.1	72.9376
GCA_965139415.1	72.9376	GCA_013149315.1	72.9371
GCA_036518795.1	72.9338	GCA_044238415.1	72.9331
GCA_034424435.1	72.9323	GCA_012275365.1	72.9296
GCA_014199635.1	72.9252	GCA_044225335.1	72.9196
GCA_035653915.1	72.9162	GCA_014194705.1	72.9148
GCA_014193435.1	72.9139	GCA_900117425.1	72.911
GCA_001296055.1	72.8937	GCA_031410735.1	72.8928
GCA_046063395.1	72.8864	GCA_031392875.1	72.8837
GCA_014194985.1	72.8816	GCA_964630805.1	72.8807
GCA_046292025.1	72.8746	GCA_002281995.1	72.8718
GCA_964413325.1	72.8597	GCA_001556015.1	72.8585
GCA_964592205.1	72.8501	GCA_031422595.1	72.84
GCA_042650965.1	72.8394	GCA_937897535.1	72.8378
GCA_012932495.1	72.8367	GCA_002278815.1	72.8343
GCA_031424035.1	72.8321	GCA_031370295.1	72.8289
GCA_035349575.1	72.8288	GCA_041639335.1	72.821
GCA_031410235.1	72.8142	GCA_046063325.1	72.8126
GCA_964245935.1	72.8052	GCA_031383985.1	72.7972
GCA_012932685.1	72.7925	GCA_001519075.1	72.7891
GCA_031420935.1	72.7876	GCA_041911195.1	72.7852
GCA_040509895.1	72.7832	GCA_027311675.1	72.773
GCA_002280995.1	72.7665	GCA_042662465.1	72.7617
GCA_007991615.1	72.7571	GCA_041639475.1	72.755
GCA_012641335.1	72.7548	GCA_001598375.1	72.7524
GCA_031846015.1	72.7407	GCA_031424455.1	72.7275
GCA_037133355.1	72.7217	GCA_937894625.1	72.7129
GCA_003249355.1	72.6994	GCA_043781375.1	72.6933
GCA_964569825.1	72.6853	GCA_009909235.1	72.6818
GCA_031417815.1	72.666	GCA_038921255.1	72.662
GCA_031411415.1	72.6616	GCA_030830245.1	72.6596
GCA_964594815.1	72.6567	GCA_017744735.1	72.651
GCA_920987055.1	72.6419	GCA_041677945.1	72.6385
GCA_020446505.1	72.6384	GCA_003534535.1	72.6352
GCA_965228995.1	72.6286	GCA_030696665.1	72.6252
GCA_031408075.1	72.6236	GCA_041677395.1	72.6131
GCA_023266625.1	72.607	GCA_038115575.1	72.6049

Continued on next page

(Continued from previous page)

Accession Code	OrthoANI (%)	Accession Code	OrthoANI (%)
GCA_965229225.1	72.6048	GCA_002256985.1	72.5966
GCA_050248535.1	72.5955	GCA_020404405.1	72.5715
GCA_031410895.1	72.5677	GCA_046716665.1	72.5601
GCA_031429755.1	72.5547	GCA_043756845.1	72.5462
GCA_031414115.1	72.5447	GCA_026400375.1	72.5413
GCA_900176395.1	72.5407	GCA_025356575.1	72.5342
GCA_026672515.1	72.5129	GCA_013824445.1	72.5121
GCA_031410955.1	72.5054	GCA_035525735.1	72.5035
GCA_945906105.1	72.497	GCA_041639295.1	72.4936
GCA_035412685.1	72.4869	GCA_002281675.1	72.4837
GCA_031369495.1	72.4805	GCA_030697365.1	72.4708
GCA_046294745.1	72.4707	GCA_031372185.1	72.4686
GCA_043717335.1	72.4661	GCA_046052745.1	72.4497
GCA_964628635.1	72.4478	GCA_017744275.1	72.4436
GCA_008015545.1	72.4323	GCA_043737775.1	72.4321
GCA_014196055.1	72.426	GCA_043738295.1	72.4244
GCA_031375005.1	72.4117	GCA_017163935.1	72.4062
GCA_046063365.1	72.387	GCA_043758555.1	72.3837
GCA_043740455.1	72.381	GCA_041639635.1	72.3802
GCA_040508185.1	72.3778	GCA_002256775.1	72.3691
GCA_002256795.1	72.3685	GCA_042652045.1	72.3667
GCA_024236245.1	72.3634	GCA_031413595.1	72.3613
GCA_019748365.1	72.3521	GCA_050172975.1	72.3476
GCA_017989315.1	72.343	GCA_049685755.1	72.3389
GCA_043761045.1	72.3375	GCA_046070565.1	72.3093
GCA_001598555.1	72.3017	GCA_050248315.1	72.3014
GCA_024236235.1	72.2852	GCA_009707465.1	72.2849
GCA_031410575.1	72.282	GCA_027486195.1	72.28
GCA_019746695.1	72.2796	GCA_031416535.1	72.2775
GCA_049240505.1	72.2735	GCA_031394575.1	72.2723
GCA_000375445.1	72.2722	GCA_964350545.1	72.2575
GCA_000192575.1	72.2509	GCA_038115065.1	72.2452
GCA_039595395.1	72.241	GCA_031428495.1	72.2401
GCA_046063275.1	72.2354	GCA_964409895.1	72.2205
GCA_031393785.1	72.2111	GCA_031415355.1	72.2087
GCA_031397605.1	72.2033	GCA_017987575.1	72.2029
GCA_046596215.1	72.1911	GCA_031367905.1	72.1784
GCA_035428385.1	72.1775	GCA_964461895.1	72.1755
GCA_044379435.1	72.1679	GCA_046590275.1	72.1652
GCA_028607105.1	72.158	GCA_050187115.1	72.1491
GCA_039928735.1	72.1434	GCA_025351285.1	72.1404
GCA_027489235.1	72.1364	GCA_040004085.1	72.135

Continued on next page

(Continued from previous page)

Accession Code	OrthoANI (%)	Accession Code	OrthoANI (%)
GCA_046155055.1	72.1287	GCA_031429135.1	72.1212
GCA_964529505.1	72.1163	GCA_028736195.1	72.1155
GCA_041915685.1	72.1126	GCA_043768005.1	72.1085
GCA_030388345.1	72.1057	GCA_031362485.1	72.1021
GCA_039925845.1	72.0949	GCA_027489515.1	72.0821
GCA_031374105.1	72.0807	GCA_020446595.1	72.0754
GCA_020446575.1	72.0533	GCA_964245645.1	72.0521
GCA_043739425.1	72.0513	GCA_043742045.1	72.0453
GCA_043761185.1	72.0383	GCA_039926895.1	72.0363
GCA_017306095.1	72.0284	GCA_043735825.1	72.0213
GCA_964582485.1	72.0206	GCA_046063295.1	72.0037
GCA_001519055.1	71.9977	GCA_039922155.1	71.9948
GCA_043767725.1	71.9819	GCA_964603645.1	71.9809
GCA_043736555.1	71.9788	GCA_043730475.1	71.9716
GCA_964609325.1	71.9688	GCA_040079735.1	71.9657
GCA_039930185.1	71.9387	GCA_043750475.1	71.9387
GCA_035428725.1	71.9301	GCA_964411455.1	71.9249
GCA_945870625.1	71.9196	GCA_014377165.1	71.911
GCA_031410255.1	71.9077	GCA_964572445.1	71.9072
GCA_009885425.1	71.9068	GCA_001725345.1	71.9059
GCA_012275515.1	71.9008	GCA_001295795.1	71.8946
GCA_031366845.1	71.886	GCA_000429005.1	71.8804
GCA_031455585.1	71.8669	GCA_039915395.1	71.8641
GCA_025354025.1	71.8522	GCA_043764355.1	71.8397
GCA_029247785.1	71.8333	GCA_043757715.1	71.8329
GCA_027490345.1	71.8206	GCA_938009165.1	71.8082
GCA_964571305.1	71.7954	GCA_040080525.1	71.7921
GCA_031418235.1	71.7884	GCA_014196525.1	71.7779
GCA_001898925.1	71.7684	GCA_031375125.1	71.7665
GCA_024642085.1	71.759	GCA_002336765.1	71.7481
GCA_013149295.1	71.7408	GCA_004005905.1	71.7335
GCA_043736815.1	71.7256	GCA_965276995.1	71.7253
GCA_043758955.1	71.7112	GCA_031365745.1	71.7061
GCA_043758655.1	71.6923	GCA_020446545.1	71.6921
GCA_043756025.1	71.6875	GCA_043768305.1	71.684
GCA_040398825.1	71.6826	GCA_045629505.1	71.6783
GCA_965214355.1	71.6716	GCA_002280675.1	71.6708
GCA_040507815.1	71.6544	GCA_964651925.1	71.6532
GCA_046594695.1	71.6377	GCA_027312145.1	71.6141
GCA_043731725.1	71.6127	GCA_001519065.1	71.6075
GCA_031419035.1	71.6072	GCA_040079535.1	71.5946
GCA_031366335.1	71.5944	GCA_031418655.1	71.5845

Continued on next page

(Continued from previous page)

Accession Code	OrthoANI (%)	Accession Code	OrthoANI (%)
GCA_039593385.1	71.5717	GCA_031372125.1	71.5592
GCA_040380165.1	71.5451	GCA_043771675.1	71.5353
GCA_031370725.1	71.532	GCA_041639435.1	71.5109
GCA_947085475.1	71.5096	GCA_031384435.1	71.5095
GCA_043770885.1	71.5076	GCA_031361075.1	71.5016
GCA_043742645.1	71.4891	GCA_026417215.1	71.4837
GCA_031370045.1	71.479	GCA_025362695.1	71.4615
GCA_031408255.1	71.4478	GCA_031410515.1	71.4323
GCA_043767865.1	71.4314	GCA_019751045.1	71.4304
GCA_031360105.1	71.4261	GCA_049243325.1	71.3416
GCA_031380975.1	71.2836	GCA_031418515.1	71.2822
GCA_043757285.1	71.2581	GCA_031427435.1	71.2255
GCA_043735705.1	71.2066	GCA_020853995.1	71.1925
GCA_046593145.1	71.1654	GCA_027486955.1	71.1315
GCA_020446445.1	71.0889	GCA_046593415.1	71.0744
GCA_031429175.1	71.069	GCA_937860195.1	71.0452
GCA_046596145.1	71.0257	GCA_031410395.1	70.9391
GCA_050175675.1	70.9196	GCA_043732365.1	70.7539
GCA_902168315.1	70.266	GCA_900218065.1	70.2592
GCA_020446465.1	69.624	GCA_964658585.1	69.5206
GCA_943371135.1	66.499	GCA_902168305.1	66.4252
GCA_964658845.1	63.209		

Table S5. Complete orthoANI Analysis of *Novosphingobium mississippiense* BL-8H^{T} (Lee et al., 2016).

Accession Code	OrthoANI (%)	Accession Code	OrthoANI (%)
GCA_044029535.1	100	GCA_044029435.1	87.5477
GCA_017309955.1	84.1395	GCA_042434475.1	84.0925
GCA_026420865.1	84.0345	GCA_943913065.1	83.9822
GCA_004341845.1	83.8734	GCA_009811895.1	81.6945
GCA_036820275.1	81.6577	GCA_032017565.1	81.5608
GCA_003241455.1	81.5393	GCA_017745295.1	81.1815
GCA_002149965.1	81.1759	GCA_032099065.1	80.953
GCA_050725775.1	80.8981	GCA_963522375.1	80.8532
GCA_009360525.1	80.8509	GCA_004368805.1	80.6733
GCA_004342985.1	80.5805	GCA_035658495.1	80.5495
GCA_044987305.1	80.4991	GCA_032093395.1	80.4862
GCA_902168295.1	80.4591	GCA_032054785.1	80.4403
GCA_004341305.1	80.2659	GCA_029239695.1	80.2211
GCA_032104605.1	80.1949	GCA_000445125.1	80.1647
GCA_001298105.1	80.1477	GCA_036352595.1	78.5078
GCA_042432915.1	78.3311	GCA_042683865.1	78.2163
GCA_022832375.1	78.2048	GCA_025153475.1	78.136
GCA_042660805.1	78.084	GCA_042682425.1	78.048
GCA_030209485.1	78.0367	GCA_035511275.1	78.0351
GCA_027922145.1	78.0088	GCA_002896965.1	78.0022
GCA_001590985.1	77.9964	GCA_050470805.1	77.9485
GCA_900113255.1	77.9427	GCA_041276605.1	77.9332
GCA_000281975.1	77.9203	GCA_021227995.1	77.8655
GCA_037384205.1	77.7903	GCA_001046635.1	77.7415
GCA_015594425.1	77.7167	GCA_001742225.1	77.6285
GCA_000632105.1	77.5669	GCA_044813085.1	77.5114
GCA_050433305.1	77.5068	GCA_022832385.1	77.4682
GCA_902706245.1	77.4488	GCA_030369695.1	77.4421
GCA_037384285.1	77.4204	GCA_902706195.1	77.34
GCA_965140495.1	77.2976	GCA_047605225.1	77.2045
GCA_000876675.2	77.1385	GCA_000235975.2	77.1102
GCA_035278205.1	77.1075	GCA_014645195.1	77.1045
GCA_001368935.1	77.0783	GCA_017312445.1	77.0258
GCA_900168325.1	76.9886	GCA_014640675.1	76.9872
GCA_000767465.1	76.9781	GCA_000802225.1	76.9406
GCA_009856825.1	76.8991	GCA_000253255.1	76.8759
GCA_042646585.1	76.7843	GCA_042661245.1	76.7704
GCA_029076845.1	76.7673	GCA_042681875.1	76.7508
GCA_900182875.1	76.6803	GCA_025340265.1	76.6696
GCA_050251905.1	76.6688	GCA_001421325.1	76.6271
GCA_044340705.1	76.5964	GCA_022832435.1	76.5334

Continued on next page

(Continued from previous page)

Accession Code	OrthoANI (%)	Accession Code	OrthoANI (%)
GCA_015865035.1	76.5095	GCA_042683985.1	76.4636
GCA_000272475.1	76.4246	GCA_014652555.1	76.4168
GCA_002684595.1	76.3996	GCA_035276965.1	76.3856
GCA_002916655.1	76.3004	GCA_041911635.1	76.2534
GCA_047246705.1	76.2349	GCA_036733805.1	76.1023
GCA_018491765.1	76.0871	GCA_018417475.1	76.0518
GCA_022818885.1	75.7779	GCA_014204535.1	75.7579
GCA_000813185.1	75.7039	GCA_020524485.1	75.6436
GCA_902506425.1	75.6339	GCA_913776895.1	75.5942
GCA_041677785.1	75.5899	GCA_002556635.1	75.3655
GCA_913777775.1	75.3449	GCA_913775085.1	75.3259
GCA_964502105.1	75.2959	GCA_913774745.1	75.2556
GCA_036384395.1	75.2286	GCA_008107685.1	75.214
GCA_913776985.1	75.1457	GCA_913778915.1	75.1454
GCA_913778265.1	75.1201	GCA_913775225.1	75.1127
GCA_913777595.1	75.1005	GCA_964277725.1	75.0854
GCA_913774105.1	75.0813	GCA_020848655.1	75.0477
GCA_014196615.1	75.0418	GCA_913777965.1	75.0158
GCA_913776335.1	75.0028	GCA_913777055.1	74.9572
GCA_913776345.1	74.95	GCA_913778115.1	74.8928
GCA_001476695.1	74.8918	GCA_913775125.1	74.8752
GCA_913777865.1	74.8383	GCA_025938095.1	74.8358
GCA_047605105.1	74.8304	GCA_913775135.1	74.8188
GCA_913778485.1	74.7957	GCA_964277855.1	74.7863
GCA_031285165.1	74.7589	GCA_964277795.1	74.6877
GCA_913777155.1	74.6467	GCA_041919975.1	74.6318
GCA_009746585.1	74.6081	GCA_014652615.1	74.5955
GCA_041912915.1	74.5747	GCA_964619635.1	74.5486
GCA_014230315.1	74.5443	GCA_027530385.1	74.5342
GCA_027532205.1	74.5288	GCA_027531025.1	74.5047
GCA_002855555.1	74.4998	GCA_004211435.1	74.4998
GCA_964520765.1	74.4997	GCA_027531865.1	74.488
GCA_913775435.1	74.4826	GCA_027530785.1	74.4734
GCA_027532715.1	74.4731	GCA_042646485.1	74.4646
GCA_027532385.1	74.4607	GCA_902168285.1	74.4514
GCA_021299075.1	74.4496	GCA_913778675.1	74.426
GCA_014230305.1	74.42	GCA_037482895.1	74.3919
GCA_027531065.1	74.3838	GCA_035350035.1	74.3821
GCA_016702815.1	74.3796	GCA_035413165.1	74.3689
GCA_046307415.1	74.3376	GCA_041912655.1	74.3327
GCA_014230345.1	74.3168	GCA_041909515.1	74.3153
GCA_014230355.1	74.2731	GCA_964561375.1	74.2699

Continued on next page

(Continued from previous page)

Accession Code	OrthoANI (%)	Accession Code	OrthoANI (%)
GCA_913778475.1	74.2544	GCA_964627175.1	74.2454
GCA_938005655.1	74.2404	GCA_046307075.1	74.2234
GCA_033242265.1	74.2214	GCA_963923805.1	74.2062
GCA_016719755.1	74.1905	GCA_035324535.1	74.1826
GCA_035281095.1	74.1817	GCA_937864195.1	74.1764
GCA_023228625.1	74.1751	GCA_035416145.1	74.1751
GCA_030699305.1	74.1733	GCA_042649005.1	74.1705
GCA_018434425.1	74.1704	GCA_041915465.1	74.1548
GCA_050281025.1	74.1425	GCA_019075875.1	74.1378
GCA_020179475.1	74.1367	GCA_041905565.1	74.1171
GCA_035423205.1	74.111	GCA_937873015.1	74.1084
GCA_001014975.1	74.1042	GCA_002279725.1	74.1036
GCA_046300735.1	74.1028	GCA_020179425.1	74.0875
GCA_015661825.1	74.087	GCA_024639725.1	74.0819
GCA_046302515.1	74.0799	GCA_028697875.1	74.0779
GCA_046339575.1	74.0716	GCA_964413885.1	74.0533
GCA_035327785.1	74.0493	GCA_041424155.1	74.0438
GCA_015390225.1	74.0333	GCA_035543295.1	74.0078
GCA_025938155.1	73.9847	GCA_938001925.1	73.982
GCA_005145025.1	73.9794	GCA_035430905.1	73.9703
GCA_964533975.1	73.9676	GCA_039927935.1	73.9517
GCA_964573075.1	73.9481	GCA_937996535.1	73.9318
GCA_039917735.1	73.9284	GCA_027489625.1	73.9109
GCA_003856955.1	73.8954	GCA_024236175.1	73.8925
GCA_040813635.1	73.8697	GCA_046052725.1	73.8587
GCA_030182715.1	73.857	GCA_964621355.1	73.8557
GCA_036383795.1	73.8432	GCA_036400975.1	73.8348
GCA_041910515.1	73.8344	GCA_046063245.1	73.8234
GCA_002813245.1	73.8149	GCA_037076535.1	73.802
GCA_015657645.1	73.7987	GCA_040534015.1	73.7981
GCA_050725835.1	73.7906	GCA_015694345.1	73.7869
GCA_038921155.1	73.7741	GCA_002279875.1	73.7594
GCA_035386545.1	73.7577	GCA_002336885.1	73.7533
GCA_046074165.1	73.7491	GCA_032337075.1	73.7436
GCA_001725355.1	73.7417	GCA_900102455.1	73.7297
GCA_029436685.1	73.7175	GCA_046306915.1	73.7093
GCA_000013325.1	73.7057	GCA_040079495.1	73.7011
GCA_002454125.1	73.6912	GCA_964343385.1	73.6788
GCA_046307775.1	73.6756	GCA_013408095.1	73.6734
GCA_040377095.1	73.668	GCA_027325615.1	73.6677
GCA_024236215.1	73.6637	GCA_035653935.1	73.6603
GCA_014640055.1	73.6554	GCA_036803765.1	73.6307

Continued on next page

(Continued from previous page)

Accession Code	OrthoANI (%)	Accession Code	OrthoANI (%)
GCA_039915025.1	73.6166	GCA_046291115.1	73.6117
GCA_031421295.1	73.6107	GCA_046063225.1	73.6098
GCA_039914785.1	73.6084	GCA_020852455.1	73.6082
GCA_012927405.1	73.6068	GCA_025938935.1	73.6044
GCA_013822565.1	73.6004	GCA_007954425.1	73.598
GCA_042685765.1	73.5924	GCA_046340395.1	73.5842
GCA_000410615.1	73.5834	GCA_041396785.1	73.5812
GCA_003171715.1	73.5756	GCA_046290865.1	73.5614
GCA_046291335.1	73.5596	GCA_041915005.1	73.5574
GCA_035571815.1	73.5552	GCA_013141325.1	73.5393
GCA_002281995.1	73.5337	GCA_035328685.1	73.5201
GCA_014652855.1	73.5191	GCA_042434435.1	73.5078
GCA_036784765.1	73.5003	GCA_964493835.1	73.4807
GCA_039543125.1	73.4745	GCA_003454795.1	73.474
GCA_046294595.1	73.4677	GCA_042648825.1	73.4572
GCA_035426945.1	73.4456	GCA_036518795.1	73.4423
GCA_043785715.1	73.4221	GCA_000807925.1	73.4215
GCA_035719485.1	73.3974	GCA_012932685.1	73.3942
GCA_050281045.1	73.3847	GCA_964573265.1	73.3816
GCA_035653915.1	73.3743	GCA_964242815.1	73.3682
GCA_014199635.1	73.3649	GCA_035345455.1	73.3646
GCA_046063265.1	73.3626	GCA_014193835.1	73.3615
GCA_046291445.1	73.3549	GCA_945906005.1	73.354
GCA_015169775.1	73.3509	GCA_003058145.1	73.3492
GCA_019510335.1	73.3467	GCA_031368085.1	73.3455
GCA_003050985.1	73.3432	GCA_003058045.1	73.3432
GCA_027340905.1	73.3426	GCA_003058095.1	73.3372
GCA_014193455.1	73.334	GCA_012641315.1	73.3283
GCA_014193715.1	73.3226	GCA_001295765.1	73.3202
GCA_002279815.1	73.3184	GCA_042662465.1	73.3169
GCA_007830315.1	73.3149	GCA_031422455.1	73.3078
GCA_037572715.1	73.3059	GCA_014194895.1	73.3039
GCA_014193435.1	73.3021	GCA_050920675.1	73.3002
GCA_964574135.1	73.299	GCA_028292865.1	73.2977
GCA_013822005.1	73.297	GCA_002440635.1	73.2963
GCA_014193535.1	73.2871	GCA_031428115.1	73.2784
GCA_035563755.1	73.2722	GCA_001590965.1	73.2707
GCA_040007615.1	73.2617	GCA_040544855.1	73.2565
GCA_013149315.1	73.2517	GCA_042650965.1	73.2488
GCA_031454595.1	73.2396	GCA_035336345.1	73.2365
GCA_014194985.1	73.2357	GCA_019748305.1	73.2332
GCA_041909635.1	73.2328	GCA_964550775.1	73.2273

Continued on next page

(Continued from previous page)

Accession Code	OrthoANI (%)	Accession Code	OrthoANI (%)
GCA_014193775.1	73.2246	GCA_014195215.1	73.2238
GCA_014194705.1	73.2214	GCA_043734515.1	73.2167
GCA_964563565.1	73.2118	GCA_001556015.1	73.2044
GCA_964468165.1	73.2041	GCA_036275395.1	73.2017
GCA_046292025.1	73.201	GCA_031392875.1	73.1979
GCA_002198665.1	73.1977	GCA_937897535.1	73.197
GCA_014195075.1	73.1969	GCA_042662505.1	73.1946
GCA_020200285.1	73.1867	GCA_034424435.1	73.1806
GCA_044238415.1	73.1789	GCA_001598575.1	73.1726
GCA_041396825.1	73.1632	GCA_964630805.1	73.1577
GCA_012275365.1	73.1541	GCA_965139415.1	73.1529
GCA_001296055.1	73.1497	GCA_020446485.1	73.1458
GCA_012932495.1	73.1312	GCA_038921205.1	73.1234
GCA_046063395.1	73.1222	GCA_041677395.1	73.1187
GCA_020446565.1	73.1175	GCA_964413325.1	73.115
GCA_014193475.1	73.1139	GCA_001598375.1	73.0956
GCA_027311675.1	73.0884	GCA_035349575.1	73.0822
GCA_041639475.1	73.0792	GCA_044225335.1	73.0644
GCA_031410735.1	73.051	GCA_031410235.1	73.0505
GCA_044378785.1	73.0447	GCA_964592205.1	73.0433
GCA_027533085.1	73.0409	GCA_027311705.1	73.0361
GCA_030830245.1	73.0327	GCA_003249355.1	73.0194
GCA_031364005.1	73.0151	GCA_041677945.1	72.9965
GCA_012641335.1	72.9948	GCA_964245935.1	72.9906
GCA_031360905.1	72.9865	GCA_031846015.1	72.9794
GCA_017744735.1	72.978	GCA_050248535.1	72.9772
GCA_046070605.1	72.9665	GCA_031422595.1	72.9624
GCA_037133355.1	72.9561	GCA_900117425.1	72.9511
GCA_041639335.1	72.9462	GCA_002278815.1	72.9439
GCA_041911195.1	72.9421	GCA_031424455.1	72.9384
GCA_046063325.1	72.9355	GCA_031420935.1	72.9276
GCA_900176395.1	72.9266	GCA_001519075.1	72.9162
GCA_031424035.1	72.9092	GCA_043756845.1	72.9074
GCA_017744275.1	72.9051	GCA_965228995.1	72.9051
GCA_026672515.1	72.8985	GCA_920987055.1	72.887
GCA_025356575.1	72.8855	GCA_965229225.1	72.8798
GCA_002256985.1	72.8648	GCA_046052745.1	72.8608
GCA_031414115.1	72.8595	GCA_002281675.1	72.8562
GCA_937894625.1	72.8474	GCA_046294745.1	72.8438
GCA_031370295.1	72.842	GCA_023266625.1	72.8362
GCA_043781375.1	72.8361	GCA_003534535.1	72.8282
GCA_002280995.1	72.8189	GCA_026400375.1	72.8178

Continued on next page

(Continued from previous page)

Accession Code	OrthoANI (%)	Accession Code	OrthoANI (%)
GCA_035412685.1	72.8011	GCA_031411415.1	72.7965
GCA_031410895.1	72.7954	GCA_009909235.1	72.795
GCA_020404405.1	72.7936	GCA_007991615.1	72.7865
GCA_013824445.1	72.7863	GCA_031417815.1	72.7815
GCA_020446505.1	72.7796	GCA_014196055.1	72.7756
GCA_039595395.1	72.7726	GCA_040509895.1	72.7718
GCA_031383985.1	72.7609	GCA_043737775.1	72.7589
GCA_030696665.1	72.7554	GCA_019748365.1	72.7321
GCA_964594815.1	72.7312	GCA_043717335.1	72.7232
GCA_031372185.1	72.7131	GCA_031410955.1	72.7126
GCA_008015545.1	72.7079	GCA_031369495.1	72.7039
GCA_049240505.1	72.7031	GCA_964569825.1	72.7006
GCA_043758555.1	72.6965	GCA_049685755.1	72.6872
GCA_017163935.1	72.6862	GCA_031408075.1	72.6798
GCA_000192575.1	72.6779	GCA_038115575.1	72.6742
GCA_002256795.1	72.6734	GCA_043740455.1	72.6725
GCA_041639295.1	72.6715	GCA_964350545.1	72.6556
GCA_041639635.1	72.6399	GCA_017987575.1	72.6362
GCA_031429755.1	72.627	GCA_050172975.1	72.6202
GCA_043738295.1	72.6154	GCA_945906105.1	72.6142
GCA_035525735.1	72.6013	GCA_030697365.1	72.5927
GCA_046063275.1	72.5887	GCA_050187115.1	72.5863
GCA_001598555.1	72.5862	GCA_046716665.1	72.5829
GCA_038921255.1	72.5825	GCA_031394575.1	72.5788
GCA_031413595.1	72.5625	GCA_027489515.1	72.557
GCA_028607105.1	72.5372	GCA_038115065.1	72.5274
GCA_031367905.1	72.5137	GCA_046070565.1	72.5086
GCA_031416535.1	72.5003	GCA_024236245.1	72.4865
GCA_024236235.1	72.4861	GCA_050248315.1	72.4826
GCA_031410575.1	72.4801	GCA_031375005.1	72.4735
GCA_043739425.1	72.4708	GCA_046155055.1	72.4686
GCA_019746695.1	72.4667	GCA_046063365.1	72.4615
GCA_027486195.1	72.4398	GCA_009707465.1	72.435
GCA_031455585.1	72.434	GCA_000375445.1	72.4288
GCA_002256775.1	72.4263	GCA_000429005.1	72.4136
GCA_964461895.1	72.4131	GCA_044379435.1	72.3956
GCA_046596215.1	72.3834	GCA_017989315.1	72.3758
GCA_001725345.1	72.3511	GCA_964628635.1	72.3472
GCA_040508185.1	72.3471	GCA_017306095.1	72.3422
GCA_043768005.1	72.3295	GCA_964609325.1	72.3268
GCA_025351285.1	72.3258	GCA_030388345.1	72.3215
GCA_964529505.1	72.3213	GCA_043761045.1	72.3172

Continued on next page

(Continued from previous page)

Accession Code	OrthoANI (%)	Accession Code	OrthoANI (%)
GCA_012275515.1	72.3108	GCA_938009165.1	72.3023
GCA_042652045.1	72.2917	GCA_043767725.1	72.288
GCA_031362485.1	72.2764	GCA_029247785.1	72.2719
GCA_039928735.1	72.2665	GCA_025354025.1	72.2646
GCA_027489235.1	72.2568	GCA_028736195.1	72.2519
GCA_031397605.1	72.2507	GCA_031428495.1	72.2454
GCA_043764355.1	72.2394	GCA_035428385.1	72.2382
GCA_041915685.1	72.2352	GCA_031393785.1	72.228
GCA_039925845.1	72.2272	GCA_031429135.1	72.2268
GCA_039922155.1	72.2245	GCA_964603645.1	72.2244
GCA_046590275.1	72.224	GCA_964571305.1	72.2176
GCA_009885425.1	72.2172	GCA_001519065.1	72.2152
GCA_964411455.1	72.2126	GCA_031418235.1	72.1995
GCA_031415355.1	72.192	GCA_001519055.1	72.1838
GCA_964409895.1	72.1835	GCA_001898925.1	72.1766
GCA_043735825.1	72.1598	GCA_964582485.1	72.1493
GCA_964572445.1	72.147	GCA_020446575.1	72.1454
GCA_031374105.1	72.1433	GCA_043742045.1	72.14
GCA_031410255.1	72.1362	GCA_040079735.1	72.134
GCA_043750475.1	72.1253	GCA_043730475.1	72.1212
GCA_020446545.1	72.1114	GCA_014196525.1	72.1088
GCA_964245645.1	72.1072	GCA_031366845.1	72.1008
GCA_039926895.1	72.0878	GCA_945870625.1	72.0848
GCA_013149295.1	72.0813	GCA_043736555.1	72.064
GCA_031375125.1	72.0554	GCA_040004085.1	72.037
GCA_043761185.1	72.0338	GCA_004005905.1	72.0303
GCA_043736815.1	72.0256	GCA_964651925.1	72.0136
GCA_031419035.1	72.012	GCA_039593385.1	72.0118
GCA_014377165.1	72.0068	GCA_046063295.1	72.0054
GCA_045629505.1	72.0032	GCA_965276995.1	72.0023
GCA_002336765.1	71.99	GCA_024642085.1	71.9864
GCA_001295795.1	71.9844	GCA_965214355.1	71.9685
GCA_027490345.1	71.9622	GCA_020446595.1	71.9522
GCA_035428725.1	71.9378	GCA_043758955.1	71.9346
GCA_040507815.1	71.9298	GCA_040398825.1	71.8776
GCA_040080525.1	71.8759	GCA_031365745.1	71.8721
GCA_031372125.1	71.8634	GCA_040380165.1	71.8437
GCA_031370045.1	71.8296	GCA_027312145.1	71.8178
GCA_043758655.1	71.8023	GCA_041639435.1	71.8018
GCA_019751045.1	71.7972	GCA_043757715.1	71.7807
GCA_046594695.1	71.7769	GCA_043756025.1	71.7743
GCA_039930185.1	71.7644	GCA_043768305.1	71.7627

Continued on next page

(Continued from previous page)

Accession Code	OrthoANI (%)	Accession Code	OrthoANI (%)
GCA_031384435.1	71.7556	GCA_031418655.1	71.7543
GCA_026417215.1	71.7539	GCA_031366335.1	71.7246
GCA_043770885.1	71.7128	GCA_002280675.1	71.7091
GCA_025362695.1	71.6805	GCA_031370725.1	71.6745
GCA_043731725.1	71.6627	GCA_043771675.1	71.6616
GCA_947085475.1	71.6574	GCA_039915395.1	71.6243
GCA_043742645.1	71.6124	GCA_031410515.1	71.6089
GCA_043767865.1	71.5887	GCA_040079535.1	71.5602
GCA_031361075.1	71.5536	GCA_031408255.1	71.5491
GCA_031418515.1	71.5404	GCA_049243325.1	71.4757
GCA_046593145.1	71.4411	GCA_020446445.1	71.427
GCA_031360105.1	71.417	GCA_031427435.1	71.4117
GCA_937860195.1	71.3602	GCA_031380975.1	71.3133
GCA_031410395.1	71.3127	GCA_043757285.1	71.308
GCA_020853995.1	71.2912	GCA_043735705.1	71.281
GCA_031429175.1	71.244	GCA_046593415.1	71.1852
GCA_043732365.1	71.0942	GCA_027486955.1	71.0714
GCA_046596145.1	71.0558	GCA_050175675.1	71.0481
GCA_902168315.1	70.5616	GCA_900218065.1	70.3834
GCA_020446465.1	70.0225	GCA_964658585.1	69.7525
GCA_943371135.1	66.7612	GCA_902168305.1	66.2037
GCA_964658845.1	63.1423		

Table S6. Comparison Against the Type-Strain Genomes via RAST Annotation.

Strains: **1** = *Novosphingobium oxfordense* BL-8A^T, **2** = *Novosphingobium mississippiense* BL-8H^T, **3** = *N. clariflavum* 164^T, **4** = *N. lindaniclasticum* DSM 25409^T, **5** = *N. guangzhouense* DSM 32207^T, **6** = *N. panipatense* SM16^T

Putative Genes	1	2	3	4	5	6
**Cofactors and vitamins**						
Biotin Biosynthesis	+	+	+	+	+	+
Thiamin biosynthesis	+	+	+	+	+	+
Heme and Siroheme Biosynthesis	+	+	+	+	+	+
Coenzyme B12 biosynthesis	+	+	+	+	+	+
Riboflavin metabolism	+	+	+	+	+	+
Flavodoxin	+	-	-	-	-	-
Pyridoxin (Vitamin B6) Biosynthesis	+	+	+	+	+	+
NAD and NADP cofactor biosynthesis	+	+	+	+	+	+
Folate Biosynthesis	+	+	+	+	+	+
Coenzyme F420 synthesis	+	+	+	+	+	+
Coenzyme A Biosynthesis cluster	+	+	+	+	+	+
Quinone cofactors	-	-	-	-	-	-
**Cell Wall and Capsule**						
Lipoprotein sorting system	+	+	+	+	+	+
Rhamnose containing glycans	+	-	-	+	+	+
Murein Hydrolases	+	+	+	+	+	+
UDP-N-acetylmuramate from Fructose-6-phosphate Biosynthesis	+	+	+	+	+	+
Recycling of Peptidoglycan Amino Acids	+	+	-	+	+	+
Gram-Positive cell wall components	-	-	-	-	-	-
**Resistance to antibiotics and toxic compounds**						
Copper homeostasis	+	+	+	+	+	+
Arsenic resistance	-	-	-	-	-	-
Cobalt-zinc-cadmium resistance	+	+	+	+	+	+
Resistance to fluoroquinolones	+	+	+	+	+	+
Beta-lactamase	+	+	+	+	+	+
Mercuric reductase	+	-	+	+	+	-
Resistance to chromium compounds	+	-	+	+	+	+
**Potassium metabolism**						
Potassium homeostasis	+	+	+	+	+	+
**Membrane Transport**						
Protein secretion system, Type I	-	-	-	-	-	-
Protein secretion system, Type II	+	+	+	+	+	+
Protein secretion system, Type III	-	-	-	-	-	-
Protein and nucleoprotein secretion system, Type IV	+	+	+	+	+	+
Protein secretion system, Type V	-	-	-	-	-	-
Protein secretion system, Type VI	-	-	-	-	-	-
Protein secretion system, Type VII	+	-	-	-	-	-

Continued on next page

(Continued from previous page)

Putative Genes	1	2	3	4	5	6
Protein secretion system, Type VIII	-	-	-	-	-	-
**Iron acquisition and metabolism**						
Siderophores	+	-	-	-	-	+
Hemin transport system	-	+	+	-	+	-
Encapsulating protein for DyP-type peroxidase and ferritin-like protein oligomers	+	+	-	+	-	+
**Motility and Chemotaxis**						
Bacterial Chemotaxis	-	-	-	-	-	-
**Secondary Metabolism**						
Auxin biosynthesis	+	+	+	+	+	+
**Nitrogen Metabolism**						
Nitrogen fixation	-	-	-	-	-	-
Nitrate and nitrite ammonification	-	+	-	-	-	-
Ammonia assimilation	+	+	+	+	+	+
Denitrifying reductase gene clusters	-	+	-	-	-	-
**Stress response**						
Osmoregulation	+	+	+	+	+	+
Glutathione: Biosynthesis and gamma-glutamyl cycle	+	+	+	+	+	+
Glutathione-dependent pathway of formaldehyde detoxification	+	+	-	+	+	+
Choline and betaine uptake and betaine biosynthesis	-	-	-	-	+	-
**Metabolism of Aromatic Compounds**						
Salicylate ester degradation	+	+	+	-	+	+
Quinate degradation	+	+	+	+	+	+
Biphenyl Degradation	+	+	-	+	+	+
p-Hydroxybenzoate degradation	+	+	+	+	+	+
Catechol branch of beta-ketoadipate pathway	+	+	+	+	+	+
Gentisate catabolism	+	+	+	+	+	+
Chloroaromatic degradation pathway	-	-	-	-	-	-
Toluene degradation	-	-	-	-	-	-
Benzoate degradation	+	-	-	-	-	+
Hydroxyaromatic decarboxylase family	-	-	-	-	-	-
Salicylate and gentisate catabolism	-	+	+	+	+	+
N-heterocyclic aromatic compound degradation	-	-	-	-	-	-
Protocatechuate branch of beta-ketoadipate pathway	-	-	-	-	-	-
Homogentisate pathway of aromatic compound degradation	-	-	-	-	-	-
Central meta-cleavage pathway of aromatic compound degradation	-	-	-	-	-	-
Cresol degradation	-	-	-	-	-	-
Aromatic amine catabolism	-	-	-	-	-	-
**Sulfur Metabolism**						
Inorganic Sulfur Assimilation	+	+	-	-	-	+
Thioredoxin-disulfide reductase	+	+	+	+	+	+
Galactosylceramide and Sulfatide metabolism	+	+	+	+	+	+

Continued on next page

(Continued from previous page)

**Putative Genes**	**1**	**2**	**3**	**4**	**5**	**6**
Alkanesulfonate assimilation	+	+	+	+	+	-
**Phosphorus Metabolism**						
Polyphosphate	+	+	+	+	+	+
**Carbohydrates Metabolism**						
N-acetylglucosamine utilization	+	+	+	+	+	+
Trehalose Biosynthesis	+	+	+	+	+	+
Glycerate metabolism	+	+	+	+	+	+
Lactate utilization	+	+	+	+	+	+
Mannose Metabolism	+	+	+	+	+	+
D-ribose utilization	+	+	+	+	-	+
D-galactonate catabolism	+	+	+	+	+	+
D-gluconate and ketogluconates metabolism	+	+	+	+	+	+
Butanol Biosynthesis	+	+	+	+	+	+
Photorespiration (oxidative C2 cycle)	+	+	+	-	+	-
Glycogen metabolism	+	+	+	+	+	+
**Miscellaneous**						
Iron-sulfur cluster assembly	+	+	+	+	+	+
Single-Rhodanese-domain proteins	+	+	+	+	+	+
Phosphoglycerate mutase protein family	+	+	+	+	+	+
Dioxygenases (EC 1.14.12.-)	+	+	+	-	+	+
Dioxygenases (EC 1.13.11.-)	+	+	-	+	+	+
Lignin degradation fragments	+	+	-	+	+	+

Table S7. Proteins References from GenBank for the Sequence Similarity Network.

Accession Number	Strain	Annotation*	Amino Acids
Standards			
AAK88039.2	*Agrobacterium fabrum* C58	bifunctional monoglycerol/glucuronosyl diacylglycerol synthase (Okino et al., 2020), (glycosyltransferase)	349
CAH08930.1	*Bacteroides fragilis* NCTC 9343	galactosyltransferase (Okino et al., 2020), (putative glycosyl transferase)	383
ACL94258.1	*Caulobacter crescentus* NA1000	Sgt1 (Stankeviciute et al., 2019), (sphingolipid glucuronosyltransferase)	455
ACL94257.2	*Caulobacter crescentus* NA1000	Sgt2 (Stankeviciute et al., 2019), (sphingolipid glycosyltransferase)	394
ATI80198.1	*Sphingobium yanoikuyae* S72	glucuronosyltransferase (Okino et al., 2020), (glycosyltransferase family 1 protein)	392
AAV90581.1	*Z. mobilis* subsp. *mobilis* ZM4	glucuronosyltransferase (Okino et al., 2020), (glycosyl transferase group 1)	394

*Annotation and source provided, GenBank annotation provided in parenthetical.

REFERENCES

Felsenstein, J. (1985). Confidence limits on phylogenies: An aprroach using the bootstrap. *Evolution* 39, 783–791

Kumar, S., Stecher, G., Suleski, M., Sanderford, M., Sharma, S., and Tamura, K. (2024). Mega12: Molecular evolutionary genetic analysis version 12 for adaptive and green computing. *Molecular Biology and Evolution* 41, msae263

Lee, I., Kim, Y. O., Park, S. C., and Chun, J. (2016). OrthoANI: An improved algorithm and software for calculating average nucleotide identity. *Int J Syst Evol Microbiol* 66, 1100–1103

Okino, N., Li, M., Qu, Q., Nakagawa, T., Hayashi, Y., et al. (2020). Two bacterial glycosphingolipid synthases responsible for the synthesis of glucuronosylceramide and α-galactosylceramide. *J Biol Chem* 295, 10709–10725

Saitou, N. and Nei, M. (1987). The neighbor-joining method: a new method for reconstructing phylogenetic trees. *Molecular Biology and Evolution* 4, 406–425

Stankeviciute, G., Guan, Z., Goldfine, H., and Klein, E. (2019). *Caulobacter crescentus* adapts to phosphate starvation by synthesizing anionic glycoglycerolipids and a novel glycosphingolipid. *mBio* 10, e00107–19

Tamura, K. and Nei, M. (1993). Estimation of the number of nucleotide substitutions in the control region of mitochondrial dna in humans and chimpanzees. *Molecular Biology and Evolution* 10, 512–526

Table S8. Pairwise Glycosyltransferase Protein Sequence Comparison.

	**Sgt1a (BL-8A)**	**Sgt1b (BL-8A)**	**Sgt2 (BL-8A)**	**Gta1 (BL-8A)**
**Sgt1a (BL-8A)**	–	42.4%	23.1%	24.8%
**Sgt1b (BL-8A)**		–	24.0%	28.5%
**Sgt2 (BL-8A)**			–	26.1%
**Gta1 (BL-8A)**				–
**Sgt1a (BL-8H)**	**92.2%**	42.1%	23.1%	24.2%
**Sgt1b (BL-8H)**	41.8%	**79.5%**	21.9%	26.2%
**Sgt2 (BL-8H)**	22.8%	22.7%	**96.6%**	25.4%
**Gta1 (BL-8H)**	25.6%	28.5%	25.9%	**90.7%**
**Sgt1 (*Caulobacter*)**	39.5%	44.4%	20.0%	25.1%
**Glt-T (*Zymomonas*)**	**62.3%**	42.1%	20.1%	25.6%
**Glt-T (*Sphingobium*)**	**68.9%**	45.6%	22.5%	25.7%
**Sgt2 (*Caulobacter*)**	22.8%	25.9%	**43.8%**	23.1%
**Glt-T (*Agrobacterium*)**	25.4%	26.1%	19.8%	**46.0%**

	**Sgt1a (BL-8H)**	**Sgt1b (BL-8H)**	**Sgt2 (BL-8H)**	**Gta1 (BL-8H)**
**Sgt1a (BL-8A)**	**92.2%**	41.8%	22.8%	25.6%
**Sgt1b (BL-8A)**	42.1%	**79.5%**	22.7%	28.5%
**Sgt2 (BL-8A)**	23.1%	21.9%	**96.6%**	25.9%
**Gta1 (BL-8A)**	24.2%	26.2%	25.4%	**90.7%**
**Sgt1a (BL-8H)**	–	41.8%	23.3%	26.3%
**Sgt1b (BL-8H)**		–	21.3%	27.2%
**Sgt2 (BL-8H)**			–	25.9%
**Gta1 (BL-8H)**				–
**Sgt1 (*Caulobacter*)**	38.0%	43.6%	20.0%	25.9%
**Glt-T (*Zymomonas*)**	**61.8%**	42.3%	19.6%	26.3%
**Glt-T (*Sphingobium*)**	**69.5%**	45.3%	22.8%	25.9%
**Sgt2 (*Caulobacter*)**	23.6%	24.7%	**43.8%**	22.9%
**Glt-T (*Agrobacterium*)**	23.9%	27.3%	20.0%	**46.8%**

Table S9. Proteins from the Type Strains Identified in the Sequence Similarity Network.

**Putative Protein Name**	**Accession Number**	**Location in Genome**	**GenBank Annotation**	**No. of Amino Acids**
Novosphingobium oxfordense BL-8A^T				
Sgt1a	XJJ66036.1	chromosome 1 (CP147005.1)	glycosyltransferase family 1 protein	383
Sgt1b	XJJ70765.1	chromosome 2 (CP147008.1)	glycosyltransferase family 1 protein	400
Sgt2	XJJ68644.1	chromosome 1 (CP147005.1)	glycosyltransferase	383
Gta1	XJJ67819.1	chromosome 1 (CP147005.1)	glycosyltransferase family 1 protein	355
Novosphingobium mississippiense BL-8H^T				
Sgt1a	XJJ58848.1	chromosome 1 (CP147001.1)	glycosyltransferase family 1 protein	383
Sgt1b	XJJ60673.1	chromosome 2 (CP147003.1)	glycosyltransferase family 1 protein	410
Sgt2	XJJ59999.1	chromosome 1 (CP147001.1)	glycosyltransferase	383
Gta1	XJJ57465.1	chromosome 1 (CP147001.1)	glycosyltransferase family 1 protein	355
